# A simulation-free constrained regression approach for flux estimation in isotopically nonstationary metabolic flux analysis with applications in microalgae

**DOI:** 10.3389/fpls.2023.1140829

**Published:** 2023-11-23

**Authors:** Anika Küken, Haim Treves, Zoran Nikoloski

**Affiliations:** ^1^Bioinformatics, Institute of Biochemistry and Biology, University of Potsdam, Potsdam, Germany; ^2^Systems Biology and Mathematical Modeling, Max Planck Institute of Molecular Plant Physiology, Potsdam, Germany; ^3^School of Plant Sciences and Food Security, The George S. Wise Faculty of Life Sciences, Tel-Aviv University, Tel-Aviv, Israel

**Keywords:** metabolic flux analysis, INST-MFA, regression, 13C labeling, algae

## Abstract

**Introduction:**

Flux phenotypes from different organisms and growth conditions allow better understanding of differential metabolic networks functions. Fluxes of metabolic reactions represent the integrated outcome of transcription, translation, and post-translational modifications, and directly affect growth and fitness. However, fluxes of intracellular metabolic reactions cannot be directly measured, but are estimated via metabolic flux analysis (MFA) that integrates data on isotope labeling patterns of metabolites with metabolic models. While the application of metabolomics technologies in photosynthetic organisms have resulted in unprecedented data from ^13^CO_2_-labeling experiments, the bottleneck in flux estimation remains the application of isotopically nonstationary MFA (INST-MFA). INST-MFA entails fitting a (large) system of coupled ordinary differential equations, with metabolite pools and reaction fluxes as parameters. Here, we focus on the Calvin-Benson cycle (CBC) as a key pathway for carbon fixation in photosynthesizing organisms and ask if approaches other than classical INST-MFA can provide reliable estimation of fluxes for reactions comprising this pathway.

**Methods:**

First, we show that flux estimation with the labeling patterns of all CBC intermediates can be formulated as a single constrained regression problem, avoiding the need for repeated simulation of time-resolved labeling patterns.

**Results:**

We then compare the flux estimates of the simulation-free constrained regression approach with those obtained from the classical INST-MFA based on labeling patterns of metabolites from the microalgae *Chlamydomonas reinhardtii*, *Chlorella sorokiniana* and *Chlorella ohadii* under different growth conditions.

**Discussion:**

Our findings indicate that, in data-rich scenarios, simulation-free regression-based approaches provide a suitable alternative for flux estimation from classical INST-MFA since we observe a high qualitative agreement ( $r_{s}=0.89$ ) to predictions obtained from INCA, a state-of-the-art tool for INST-MFA.

## 1 Introduction

Rates or fluxes of biochemical reactions shape the pools of metabolites that perform diverse cellular functions, from serving as building blocks of cellular components to performing signaling functions. While metabolite concentrations and contents can be measured at cell- and (sub)compartment-specific level (Dietz, 2017; Lanekoff et al., 2022), reaction fluxes are estimated by metabolic flux analysis (MFA). MFA integrates labeling patterns of metabolites into metabolic network models, often assuming metabolic steady state, whereby pool sizes of metabolites do not change over time (Basler et al., 2018; Long and Antoniewicz, 2019; Kruger and Ratcliffe, 2021). The labeling patterns are obtained from laborious experiments in which (positionally) labeled substrates are fed to the studied biological system (e.g. cell culture or an entire organism). The incorporation of the label in the internal metabolites is then quantified either in one snapshot, suitable for studies of isotopic steady states, or over a time course, allowing the monitoring of isotopic nonstationary state. The latter allow monitoring of the evolution of label incorporation into metabolic pools. As a result, there are two classes of MFA approaches - stationary and nonstationary - that lead to different experimental and computational advantages and challenges in their application.

Irrespective of the type of MFA applied, the labeling patterns can be summarized in different forms [e.g. isotopomers (Wiechert et al., 1999), cumomers (Wiechert et al., 1999), mass isotopomer distributions (MIDs) (Antoniewicz et al., 2007)], and their evolution (and steady states) is described by a system of ordinary differential equations (ODEs) obtained by using atom transition mappings (Huß et al., 2022). These equations can be employed to simulate labeling patterns given a (steady-state) flux distribution along with initial labeling state and/or pool sizes of the considered metabolites. The problem of flux estimation is then tantamount to estimating a flux distribution for which the simulated labeling patterns are an acceptable fit for the experimentally measured. The acceptability of the fit is assessed by a goodness-of-fit measure of choice. To this end, algorithmic advances have led to decreasing the number of simulated variables [e.g. via elementary metabolite units (EMUs)] and expanding the applicability to networks of large size (Gopalakrishnan et al., 2018; Hendry et al., 2020). This workflow for flux estimation from so-called *global* MFA approaches is well established even for the isotopic nonstationary state and implemented in toolboxes like INCA (Young, 2014). However, the bottleneck remains the comparison of flux estimations from multiple model scenarios. This is largely due to the massive systems of ODEs and corresponding optimization problems whose solution to global optimality remains problematic.

One way to address this issue is to move away from estimating steady-state flux distributions for the entire network, and instead to rely on local approaches. In their simplest form, local approaches allow the estimation of flux ratios around a metabolite of interest given the labeling patterns [in the form of MIDs for the metabolite and its precursors (Hörl et al., 2013)]. With data about the evolution of all labeling patterns appearing for a considerably smaller system of ODE, the problem of flux estimation can be cast as simulation-free constrained regression (SFCR), where constraints correspond to the relationship between fluxes at steady state.

This idea readily extends to any subnetwork for which there are sufficient data about the labeling patterns of the metabolites involved. One such system for which the MIDs of practically all involved intermediates can be measured includes the Calvin-Benson cycle (CBC) and related canonical processes, namely starch and sucrose syntheses. Estimating fluxes in the CBC and other downstream processes in metabolic networks of photosynthetic organism in photoautotrophic growth conditions necessitates the usage of isotopically nonstationary MFA (INST-MFA). This is the case since under photoautotrophic growth conditions, feeding 13CO_2_ leads to an isotopic steady state that is not informative for flux estimation. As a result, relying on an approach formulated in terms of constrained regression can greatly simplify the flux estimation for metabolic processes in photosynthesis, like the CBC, and can also help investigate the effects of different model structures often used for flux estimation. As a result, the approach is also readily applicable to plants provided enough data on the labeling kinetics of metabolites in the metabolic process of interest.

Here we show that under certain mild assumptions, fluxes in the CBC can be estimated based on a SFCR approach. We used this formulation to compare the flux estimates from two model scenarios, with and without consideration of pool compartmentation, allowing further generalization of the approach at little computational cost. Our results demonstrate that fluxes from the easy-to-implement and solve SFCR are in high qualitative agreement with estimates from the state-of-the-art tool for nonstationary ^13^C-MFA, INCA (Young, 2014). However, we also find that SFCR is more sensitive to fitting of exchange fluxes, since it does not introduce multiple scaling parameters of the MIDs which are optimized during flux estimation as implemented in INCA. Given the recent increased interest in flux estimation from nonstationary ^13^C-MFA (Fu et al., 2022; Koley et al., 2022), the analysis points at factors that need to be considered and carefully compared when presenting the findings from flux estimations based on isotopic nonstationary experiments.

## 2 Results

### 2.1 Flux estimation from ^13^C labeling data using a SFCR approach

**Figure 1:**
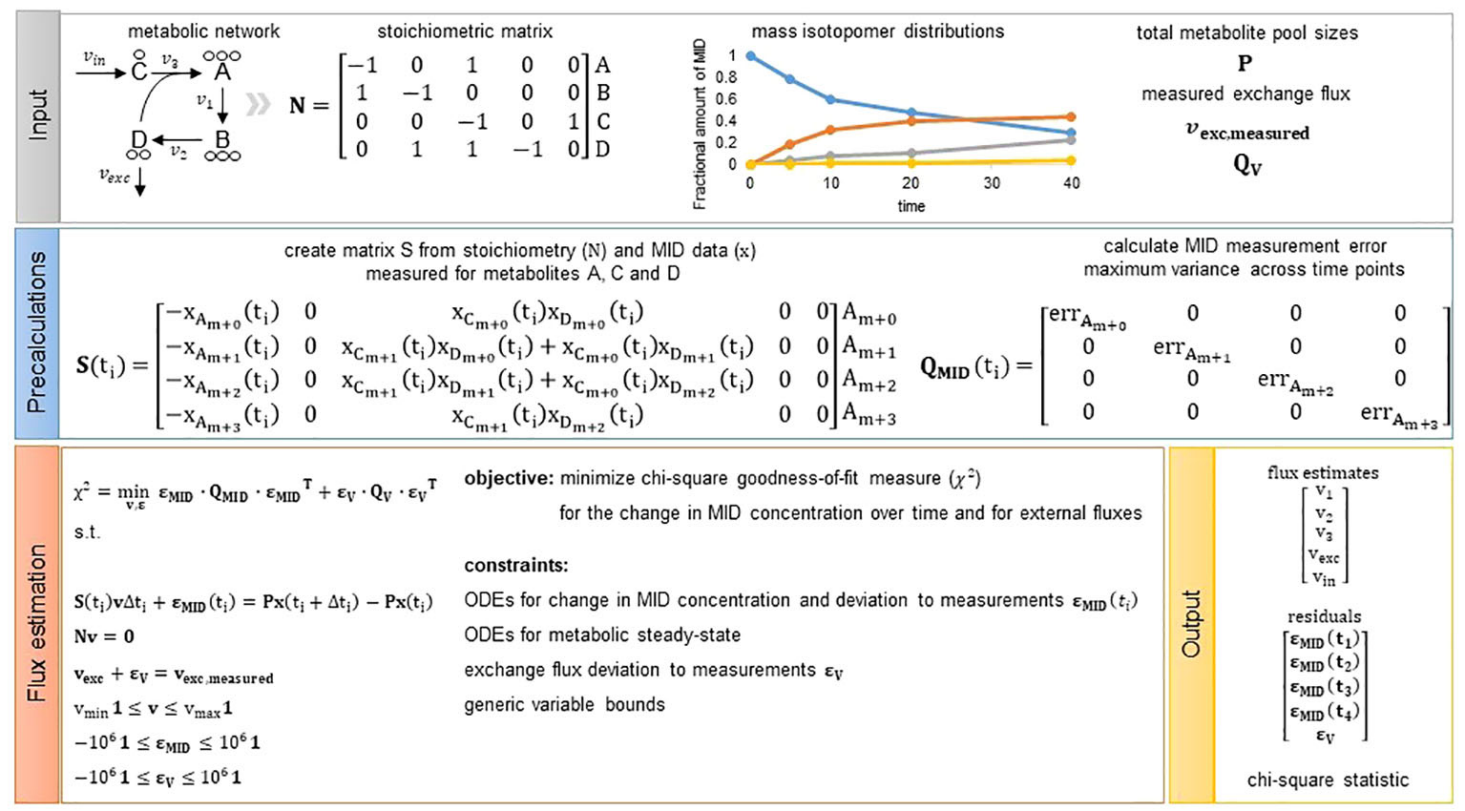
Overview of the presented flux estimation approach. Input: As input we require the structure of a metabolic network represented by a stoichiometric matrix N . The toy metabolic network consists of four metabolites **(A–D)** and five reactions ( $v_{1},v_{2},v_{3},v_{in},v_{exc}$ ). In addition, we need measurements of mass isotopomer distributions, total metabolite pool sizes for metabolites with fitted change in MID concentration (optional, see Methods Section 4.6) as well as measurements of exchange fluxes (optional) from which we can directly assign $v_{exc,measured}$ and $Q_{V}$ including the mean and variance of the measured fluxes, respectively. Precalculations: Given the network structure and MID data we can build matrix S for each measurement time point. Matrix ${\text{Q}}_{\text{MID}}$ is a diagonal matrix including the measurement error chosen to be the maximum variance observed for a MID across all time points. Hence, ${\text{Q}}_{\text{MID}}$ is not dependent on time and is the same for each time point. Flux estimation: We solve the presented quadratic program and obtain flux estimates for all model reactions, residuals to the measurements integrated and the chi-square goodness-of-fit measure used to assess quality of the fit.


Based on a metabolic network, described by the stoichiometric matrix $N\in {\mathbb{R}}^{m\times r}$ , and given measurements of metabolite pool sizes, gathered in a diagonal matrix $P\in {\mathbb{R}}^{k\times k}$ , for all metabolites with available fractional amount of measured MIDs, denoted by $x\in {\mathbb{R}}^{k\times 1}\;$ whose appropriate combinations are gathered in a matrix $S\in {\mathbb{R}}^{k\times r}$ , one can write the system of ODEs for the fractional amount of measured MIDs as follows:


$$P\frac{dx}{dt}=Sv,$$

where $v\in {\mathbb{R}}^{r\times 1}$ is the vector of fluxes we aim to estimate. For an example of how S looks like for the two CBC models, consult the **Supplementary Material** and implementation available on GitHub, the matrix S for a toy example is shown in **Figure 1**. This ODEs can be discretized following Forward Euler approximation as follows:

$$Px\left(t_{i}+\Delta t_{i}\right)-Px\left(t_{i}\right)=S\left(t_{i}\right)v\Delta t_{i},$$

corresponding to a system of linear equations. The approximation error by the Forward Euler method is in the order of $\Delta t_{i}$ and hence, sufficiently small time steps should be considered in the analyzed labeling experiment. The flux distribution, v , does not depend on time, since we assume, as in other MFA approaches, that the network is in a metabolic steady state.

Let $\varepsilon_{MID}\left(t_{i}\right)\in {\mathbb{R}}^{k\times 1}$ denote the deviation between measured and predicted change in concentration of isotopomers obtained by multiplication of metabolite pool size P and fractional amount of MIDs $x\left(t_{i}\right).$ Further, let $Q_{MID}\left(t_{i}\right)\in {\mathbb{R}}^{k\times k}$ represent a diagonal matrix that contains the variance of the change in isotopomer concentration between two time points $t_{i}$ and $t_{i}+\Delta t_{i}$ , i.e. $Y\left(t_{i}\right)=Px\left(t_{i}+\Delta t_{i}\right)-Px\left(t_{i}\right).$ Assuming that $Px\left(t_{i}+\Delta t_{i}\right)$ and $Px\left(t_{i}\right)$ are independent random variables, both of variance $\sigma^{2}$ , it follows that the variance of $Y\left(t_{i}\right)$ is $2\cdot \sigma^{2}$ . In this study, we assume that measurement error, σ , does not depend on time (see Materials and Methods); therefore, $Q_{MID}$ will not differ between time points. In addition to these constraints, we also use measurements of particular exchange fluxes to further constrain the feasible flux distributions. Let $\varepsilon_{V}\in {\mathbb{R}}^{2\times 1}$ denote the deviation between measured and estimated exchange fluxes and $Q_{V}\in {\mathbb{R}}^{2\times 2}$ be a diagonal matrix that contains the variance for the measured exchange fluxes.

Based on these simplifying assumptions, flux estimation corresponds to the following quadratic programming problem (QP) that can be regarded as a constraint-regression problem in which we minimize the chi-square goodness-of-fit measure for the change in MID concentration over time as well as external fluxes. The minimization is performed under the constraint that the entire system, represented by stoichiometric matrix N , is at metabolic steady state (see **Figure 1** for a graphical overview of the workflow for the presented approach):


$$\underset{v,{\text{ε}}_{MID},{\text{ε}}_{V}}{\text{min }}{\text{ε}}_{MID}\cdot Q_{MID}\cdot {\text{ε}}_{MID}{}^{T}+{\text{ε}}_{V}\cdot Q_{V}\cdot {\text{ε}}_{V}{}^{T}$$

s.t.

$$S\left(t_{i}\right)v\text{Δ}t_{i}+{\text{ε}}_{MID}\left(t_{i}\right)=Px\left(t_{i}+\text{Δ}t_{i}\right)-Px\left(t_{i}\right)$$

Nv=0

$$v_{exc}+{\text{ε}}_{V}=v_{exc,measured}$$

$$v_{min}1\leq v\leq v_{max}1$$

$$-10^{6}1\leq \varepsilon_{MID}\leq 10^{6}1$$

$$-10^{6}1\leq {\text{ε}}_{V}\leq 10^{6}1\;\;\;\;\;,$$

with $v_{max}=10^{6}\;nmol\;gDW^{-1}s^{-1}$ , $v_{min}=-10^{6}\;nmol\;gDW^{-1}s^{-1}$ for reversible reactions, and 0 otherwise.

### 2.2 Validation of assumptions and model set-up for estimation of fluxes in the CBC

To compare the findings of our SFCR approach with those from the state-of-the-art tool INCA, using an EMU-based global approach, we employed data on MIDs from three different algae previously used for flux estimation (Treves et al., 2021). Since we have access to the majority of MIDs of metabolites that participate in the CBC cycle, we formulate constraints for the MIDs of seven metabolites measured in *Chlamydomonas reinhardtii* and *Chlorella sorokiniana* under low light (LL, 100 µmol photons m^-2^ s^-1^), and in *Chlorella ohadii* under LL and extreme illumination levels (EIL, 3000 µmol photons m^-2^ s^-1^). Like in Treves et al. (Treves et al., 2021), we also assume that: (i) the structure of the CBC in the considered algae is the same, (ii) there are no microcompartmentation effects on the MIDs of ribulose-1,5-bisophosphate (RuBP), (iii) dihydroxyacetonphosphate (DHAP) is the predominant form of triose phosphates (T3P), hence, its labeling follows the DHAP labeling measured, and (iv) the labeling of ribulose-5-phosphate follows the labeling of pentose phosphates (PP) measured.

**Figure 2:**
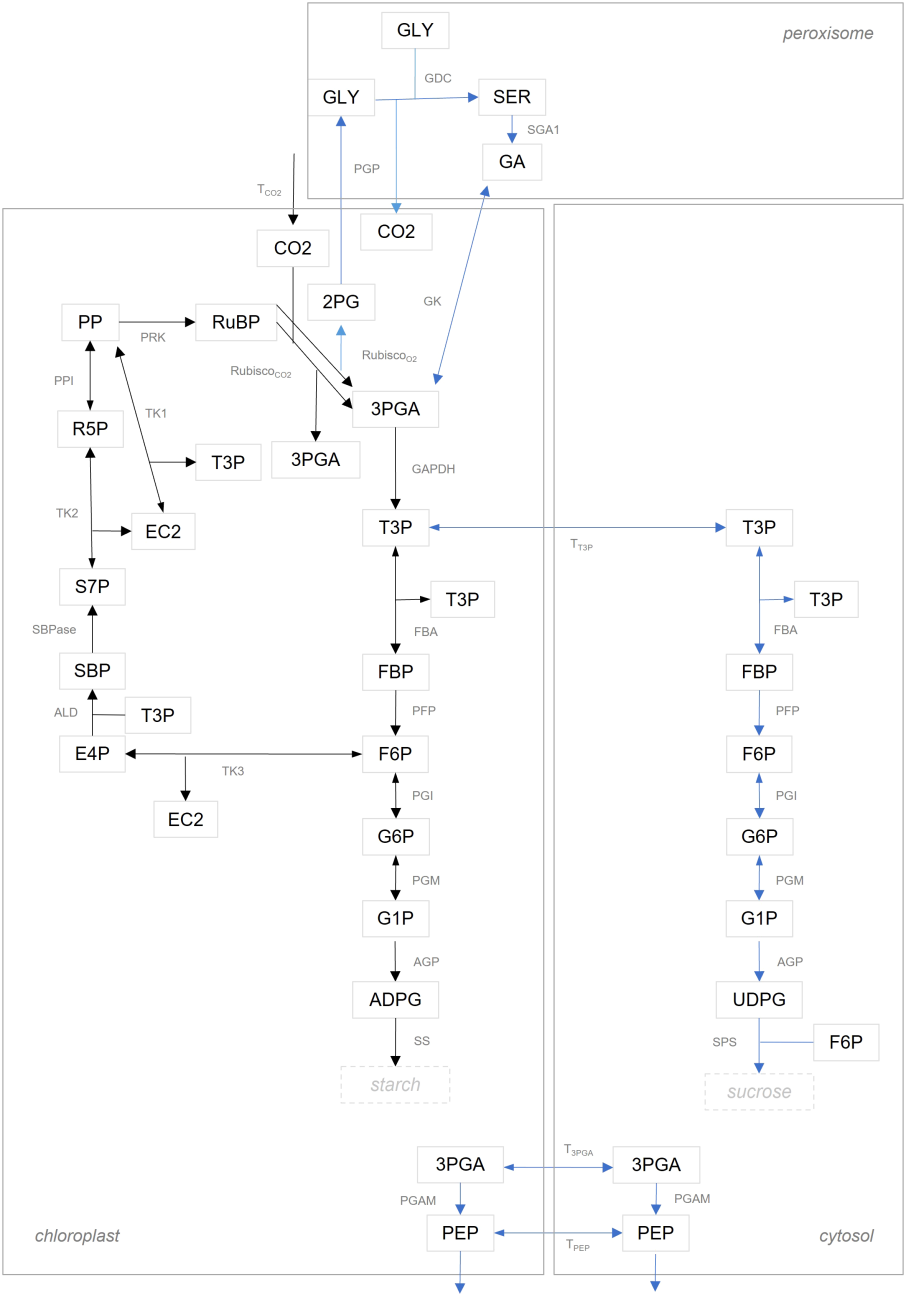
Graphical illustration of model structure. Reactions in the ‘simple CBC model’ are shown with black arrows. The set of reactions in the ‘compartmented CBC model’ are the union of reactions illustrated by black and blue arrows. A list of abbreviations used can be found in **Supplementary Information 1.2**.

We compared flux estimates resulting from integration of data about MIDs in two model variants of different complexity. The model denoted as ‘simple CBC model’, includes reactions of the CBC with only a branch towards starch synthesis; further, metabolites in the simple CBC model are not compartmentalized between the chloroplast and cytosol. The ‘simple CBC model’ included ODEs that describe the nonstationary ^13^C labeling of RuBP, F6P, FBP, G1P, G6P, and ADPG by using the MIDs of PP, RuBP, DHAP, FBP, F6P, G1P, G6P, and ADPG (see **Supplementary Information 1.1** for a full list of model reactions and ODEs as well as the full names of the metabolites, and **Figure 2** for a graphical model representation). The second model denoted as ‘compartmented CBC model’, includes reactions of the CBC, photorespiration, starch and sucrose syntheses, and considers metabolite compartmentation between chloroplast and cytosol. Based on the structure of the ‘compartmented CBC model’, in addition to the ODEs for MIDs of RuBP, F6P, FBP, G1P, G6P, ADPG, we also formulated ODEs for the MIDs of UDPG (see **Supplementary Information 1.2** for a full list of model reactions and ODEs and **Figure 2** for a graphical model representation).

Since the proposed estimation of fluxes using SFCR, like other MFA approaches, assumes metabolic steady state, we next determined if this assumption held for the metabolites whose MIDs are described in the model of the investigated algae species. To this end, we conducted ANOVA with the metabolite pool sizes over time. Our findings indicated that RuBP, FBP and ADPG were not at steady state over the investigated time course of *C. reinhardtii*. For *C. ohadii* under EIL, ADPG was the only metabolite not at steady state, while for *C. sorokiniana* all metabolites were found to be at steady state (**Supplementary Table S1**). Since steady state was ensured for the majority of metabolites, we enforced this constraint in flux estimation. In contrast, for *C. ohadii* under LL, we found RuBP and PP to be the only metabolite pools at steady state over the entire time course of the measurement. However, if we take out the last time point (40s) FBP, ADPG and UDPG were the only metabolites not at steady state (**Supplementary Table S1**). Therefore, we only used the first four time points (0s, 5s, 10s and 20s) for flux estimation in *C. ohadii* under LL.

### 2.3 Goodness-of-fit values for models and data sets from the investigated algae

With these assumptions, we cast the problem of flux estimation as a quadratic optimization problem in which we minimized the chi-squared goodness-of-fit statistic ( $\chi^{2})$ for the considered MIDs along four time points (three time points in case of *C. ohadii* under LL) as well as flux values for starch and sucrose synthesis reactions (see Materials and Methods). This approach was possible since all MIDs included in the ODEs were measured (**Supplementary Table S2**). To determine robustness of flux estimates we calculate confidence intervals (CI) for all reaction fluxes (see Methods Section 4.4, **Supplementary Table S3, S4, S8**) and report $\chi^{2}$ for the best fit and CI.

*Simple CBC model* At a significance level α=0.05 , fitting the MIDs for six metabolites and starch synthesis rate using the ‘simple CBC model’ resulted in a system with 149 degrees of freedom for which the expected range of $\chi^{2}$ , the range where $\chi^{2}$ is considered statistically acceptable (Young, 2014), is [117.1, 184.7]. For *C. reinhardtii* and *C. sorokiniana* the $\chi^{2}$ was 87.4 and 85.7 with CI [84.7, 108.6] and [78, 96.5], respectively. Since the obtained $\chi^{2}$ values were smaller than the lower bound of the interval for the expected $\chi^{2}$ , there may be an issue of over-fitting. Nevertheless, since we wanted to compare flux estimates across algae under matching set of assumptions, we used the corresponding flux estimates in the comparative analysis. The $\chi^{2}$ for *C. ohadii* under LL and EIL were 159.7 (with 108 degrees of freedom, since the last time point was not considered) and 520.6 (149 degrees of freedom), respectively, and were not considered statistically acceptable. Since the relative error in measurements under EIL is not significantly lower than in the other algae, there is no experimental basis for omitting the last time point, as there was for *C. ohadii* under LL. Our previous modeling efforts with INCA did not find acceptable fit for the data of *C. ohadii* under EIL (Treves et al., 2021). Possible differences in the model structure for *C. ohadii* under EIL provides one explanation for the discrepancy.

*Compartmented model of CBC and related pathways* At a significance level α=0.05 , fitting the MIDs for seven metabolites using the ‘compartmented CBC model’ variant as well as starch and sucrose synthesis rates resulted in a system with 166 degrees of freedom for which in the expected range of the $\chi^{2}$ is [132.2, 203.6]. Parameters modeling compartmentation were fixed to random numbers sampled from the interval [0.1, 0.9], with 50 repetitions. We reported the parameterization of c that resulted in the best fit; this may not correspond to the global optima, over all possible parameterizations for c , which is anyhow difficult to ensure for such nonlinear optimization problems. Following this procedure, we found parameterization of c for which the $\chi^{2}$ CI for *C. reinhardtii* and *C. sorokiniana* were not above the expected range with [87, 111] and [110, 132.3], respectively. In case of *C. ohadii* under LL, we fit a system with 118 degrees of freedom; therefore, at the significance level α=0.05 , the expected range of $\chi^{2}$ that would allow to accept model fit is [89.8, 150]. However, the CI for the $\chi^{2}$ of fit to labeling patterns of *C. ohadii* was [121.1, 163.7], i.e. the upper bound of the CI was above the expected range. However, the majority (81.6%) of bootstrap samples were within the acceptable range (**Supplementary Figure S1**); and therefore, we used the flux estimates for comparison across algae. The $\chi^{2}$ for the fit of ^13^C labeling data from *C. ohadii* under EIL was 598.6 and hence is not statistically acceptable. Hence, like above, it is likely that modification of the model structure may be required to model labeling dynamics in *C. ohadii* under EIL.

### 2.4 Effects of model complexity on estimated fluxes

**Figure 3:**
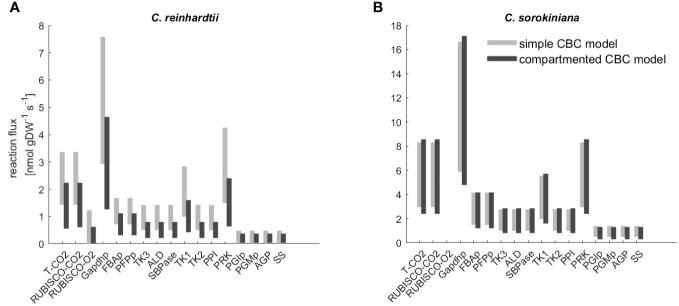
Flux range of Calvin-Benson cycle (CBC) reactions estimated from simulation-free constrained regression using models of different complexity. Flux ranges are estimated using the ‘simple CBC model’, (see **Figure 1** and **Supplementary Information 1.1** for the list of reactions and long names of reaction abbreviations used on x-axis) and the compartmented model of CBC and related pathways (‘compartmented CBC model’, see **Figure 1** and **Supplementary Information 1.2** for a list of reactions) for **(A)** *C. reinhardtii* and **(B)** *C. sorkiniana*.

Both model variants allowed us to fit labeling kinetics for *C. reinhardtii* and *C. sorokiniana*. Next, we compared the flux estimates to investigate the effects of model structure used in the fitting. We found that CI for the flux estimates of the reactions in the CBC overlap for the majority of reactions (**Figure 3**, **Supplementary Table S3**). In addition, mean flux values were highly correlated with $r_{P}=0.995$ (Spearman $r_{S}=0.998$ ) for *C. reinhardtii* and $r_{P}=1$ (Spearman $r_{S}=1)$ for *C. sorokiniana*. As a result, we concluded that the complexity of the model had only a slight effect on the estimated flux ranges with the labeling patterns of *C. reinhardtii* and *C. sorokiniana*.

### 2.5 Comparison of flux estimates across algae

**Figure 4:**
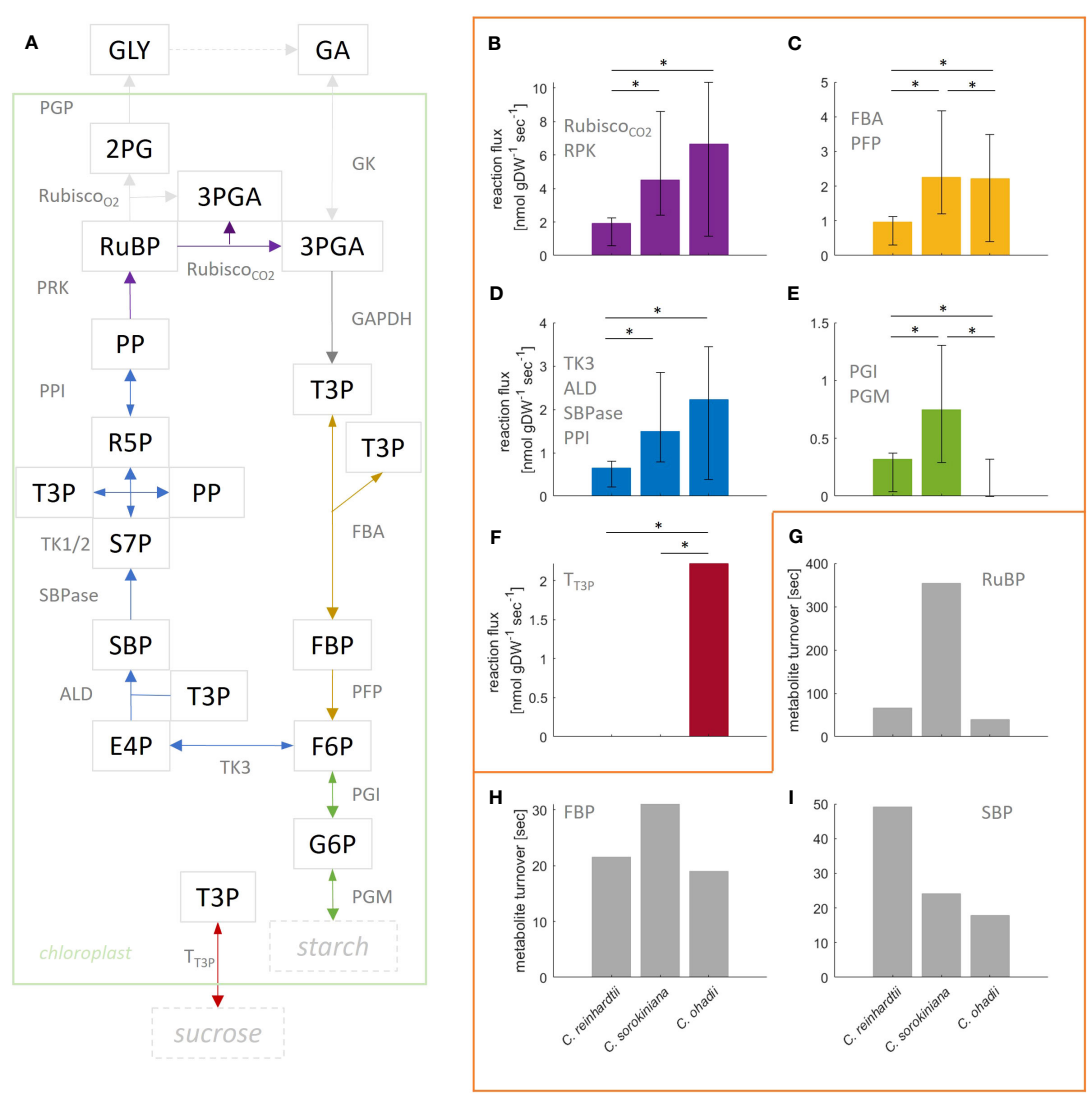
Flux estimation for reactions in the Calvin-Benson cycle (CBC) from mass isotopomer distributions using a ‘compartmented CBC model’ for three model species grown under low light conditions. **(A)** Simplified illustration of underlying CBC model. Reactions catalyzed by enzymes PGK, GAPDH and TPI are lumped and convert 3PGA into T3P. Based on the model, reactions of same color carry the same steady-state flux. For a full list of reactions and abbreviations see **Supplementary Information 1.2**, for graphical representation of the entire model see **Figure 1**. **(B–F)** Estimated flux and 95% confidence interval obtained from bootstrapped sampling indicated by error bars for the respective group of reactions; asterisk indicate significant difference in flux (p-value< 0.05); **(B)** Rubisco carboxylation (deep purple). Oxygenation was estimated to be zero for all algae and therefore, is not shown. In addition, note that GAPDH flux is twice the flux through Rubisco carboxylation and, therefore, is not shown in a separate panel; **(C)** FBA and PFP; **(D)** TK3, ALD, SBPase and PPI; **(E)** PGI and PGM; **(F)** Transport of T3P towards cytosol for sucrose production. **(G–I)** Turnover of metabolites **(G)** RuBP, **(H)** FBP and **(I)** SBP calculated from the average measured pool size and flux through reactions PRK, PFP and SBPase, respectively.

Given that the complexity of the model resulted in small effects on the flux estimates and the goodness-of-fit values, here we provide a more detailed comparative analysis using the more complex, compartmented CBC model across the three algal species under LL. *C. ohadhii* is the fastest growing green algae known to date, with high photoprotection capacity that facilitates growth under extreme light conditions (Ananyev et al., 2017). Hence, identification of differential fluxes in comparison to algae with slow growth rates provides the potential to understand the underlying molecular mechanisms that underpin the capacity for fast growth (Treves et al., 2021). We found that the estimated mean flux of the carboxylation reaction of RuBisCO under LL condition was the largest in *C. ohadii* ( $6.7\;nmol\;gDW^{-1}s^{-1}$ ), followed by *C. sorokiniana* (4.5 $\;nmol\;gDW^{-1}s^{-1}$ ) and *C. reinhardtii* ( $1.9\;nmol\;gDW^{-1}s^{-1}$ ) (**Figures 4A, B**). The mean values for RuBisCO carboxylation were in the expected order across algae, as were the upper bounds of the 95% CI. In line with this observation, previous studies found high photosynthetic rates for *C. ohadii* (Treves et al., 2016).

However, it must be also noted that the CI for *C. sorokiniana* and *C. ohadii* overlap (**Figures 4A, B**) and the difference between the fluxes was not statistically significant (t-test p-value=0.89). The mean estimated flux through RuBisCO oxygenation reaction was zero for all algae. In line with the observation for RuBisCO carboxylation, mean flux through SBPase is highest in *C. ohadii* followed by *C. sorokiniana* and *C. reinhradtii* (**Figures 4A, D**). However, the increase in flux for *C. ohadii* in comparison to *C. sorokiniana* was not significant (t-test p-value=0.08). For reactions PFP/FBA (**Figures 4A, C**), we found the smallest flux for *C. reinhardtii*. While the estimated mean flux of PFP/FBA in *C. sorokiniana* and *C. ohadii* were similar, flux sampled by the bootstrap procedure indicated that PFP flux could have significantly larger values in *C. sorokiniana* than *C. ohadii*, as observed in the CI. Measured starch synthesis rates for the three algae under LL (Treves et al., 2021) showed that starch synthesis rate was increasing with increasing photosynthesis rate and hence, growth. Moreover, the measured sucrose rates indicated a lack in sucrose production for *C. reinhardtii*, while both *C. sorokiniana* and *C. ohadii* showed similar levels of sucrose synthesis. Using these measurements to fit external fluxes in the QP, we would expect reactions PGM and PGI, the first steps in starch synthesis from CBC intermediates, to show the highest flux in *C. ohadii*, followed by *C. sorokiniana* and *C. reinhadtii* (**Figures 4A, E**). However, we observed the highest flux towards starch synthesis in *C. sorokiniana* and zero mean flux in *C. ohadii*. Flux towards sucrose synthesis was found not to be zero only for *C. ohadii* (**Figures 4A, F**). While the absence of sucrose synthesis flux is expected form experimental data integrated in the QP for *C. reinhardtii*, this is not the case for *C. sorokiniana*. In addition, it must be noted that the estimated flux values for starch and sucrose synthesis rate are two orders of magnitude smaller in comparison to the measured. The work of Treves et al. (2021) indicated that flux through PEP is differential in *C. ohadii* compared with the other algae and might also explain differences in growth. However, our analysis was not able to predict these differences. The reason for this lies in the missing coverage of MIDs in the TCA cycle as well as MIDs of PEP needed to correctly predict this flux.

In line with the findings on individual reaction level (e.g. RuBisCO carboxylation, PFP/FBA) the sum of flux through the entire CBC was the highest for *C. sorokiniana* and the smallest for *C. reinhardtii* (**Supplementary Table S4**). However, photosynthesis rates measured for the three algae suggest highest flux in *C. ohadii* (Treves et al., 2021). For a detailed examination of possible reasons, we point the reader to the Discussion.

Considering the ordering of fluxes based on their magnitudes, we found a very high correlation (Spearman, $r_{S}=1$ ) between mean flux of *C. reinhardtii* and *C. sorokiniana*. Interestingly, the Spearman correlation of the flux distribution for *C. ohadii* to that of either *C. reinhardtii* or *C. sorokiniana* was smaller ( $r_{S}=0.75$ , in both cases). The decrease in correlation for the flux distribution of *C. ohadii* with those of the other algae can be explained by the complementary pattern of carbon partitioning between starch and sucrose syntheses; specifically, in *C. ohadii* there was only positive flux towards sucrose synthesis, but no flux through starch synthesis, in contrast to the findings for *C. reinhardtii* and *C. sorokiniana* (**Figures 4E, F**). For a detailed examination of possible reasons, we point the reader to the Discussion.

We also determined the turnover times for three modeled metabolites (**Figures 4G–I**; **Supplementary Table S5**). Using the measured pool sizes, we found that the turnover of RuBP was the fastest in *C. ohadii* (40s), followed by *C. reinhardtii* (66s) and *C. sorokiniana* (6min). In line with the observed turnover of RuBP, we found that turnover of FBP was the fastest in *C. ohadii* (15s), followed by *C. reinhardtii* (22s) and *C. sorokiniana* (31s). However, while SBP exhibited the fastest turnover in *C. ohadii* (18s), *C. sorokiniana* (24s) showed similar turnover as *C. ohadii*, followed by *C. reinhadtii* (49s) with the slowest turnover for SBP. Together, these findings indicated that *C. ohadii* has more efficient photosynthesis in comparison to *C. reinhardtii*, indicated by higher carboxylation rates in comparison to *C. reinhardtii*, while metabolites pool sizes are in the same order of magnitude for both algae. In addition, it can be also concluded that *C. ohadii* has more efficient photosynthesis in comparison to *C. sorokiniana*, indicated by similar carboxylation rates for *C. ohadii* in comparison with *C. sorokiniana*, despite pool sizes in *C. ohadii* which are one order of magnitude smaller than those observed for *C. sorokiniana* (**Supplementary Table S7**). In addition, *C. ohadii* has a faster turnover of metabolite pool sizes in comparison to the other algae.

### 2.6 Comparison to flux estimates obtained from INCA

To compare the estimates from the SFCR approach to those from global MFA flux estimation, we used the state-of-the-art tool INCA (Young, 2014) with the ‘compartmented CBC model’. In addition to MID entering the SFCR formulation, the underlying model structure in INCA allowed us to use the MIDs for 3PGA, SBP, S7P, R5P, PEP and 2PG in flux estimation. Therefore, we estimated fluxes using INCA in two scenarios using: (1) MIDs fitted in the SFCR formulation and (2) all MIDs that can be used in INCA. We opted to include MIDs for more metabolites with the aim of increasing the precision of the estimates (expected due to the usage of more data). Altogether, the flux estimation included fitting of pool size, starch and sucrose synthesis rates as well as data on the aforementioned metabolite MIDs for both approaches. For a comparison of fitted labelling pattern obtained by SFCR and INCA see **Supplementary Figures S3–S5**.

**Figure 5:**
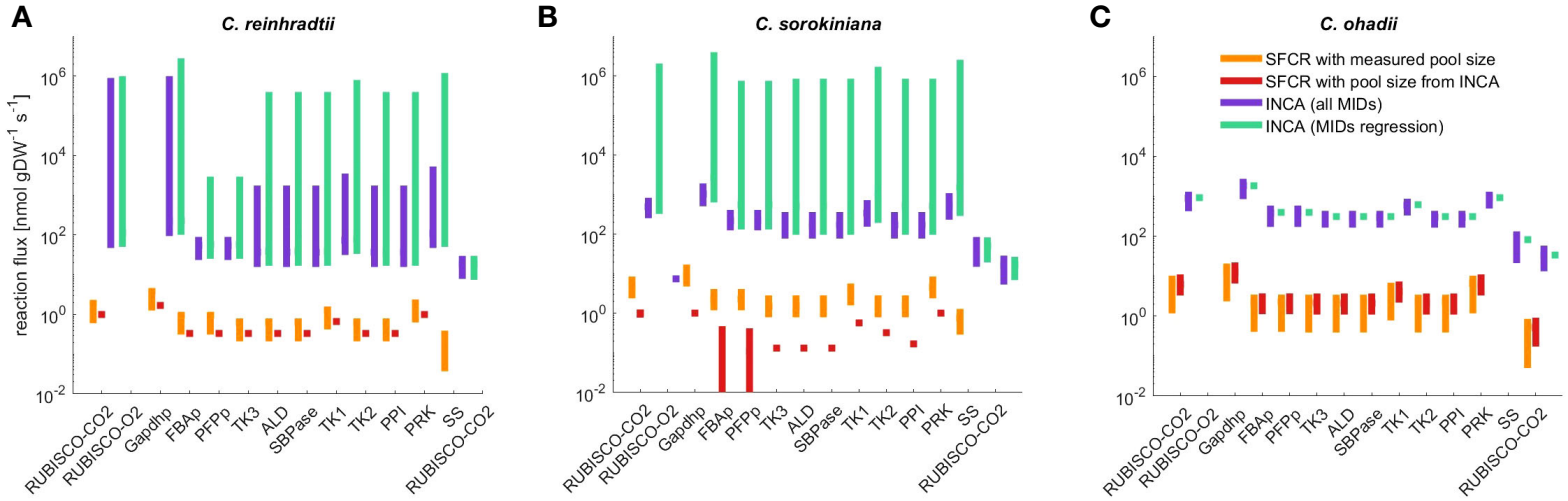
Flux range of Calvin-Benson cycle reactions, starch (SS) and sucrose synthesis (SPS) estimated from simulation-free constrained regression (SFCR) and INCA. The estimated fluxes for **(A)** *C. reinhardtii*, **(B)** *C. sorokiniana* and **(C)** *C. ohadii* from the presented SFCR approach (orange bars) are in high qualitative agreement with estimations from INCA (**Supplementary Table S9**), but lack quantitative agreement. To test if differences in metabolite pool size can explain the difference in flux between constrained regression and INCA, we calculate flux from SFCR integrating pool size estimates from INCA (red bars). See **Supplementary Information 1.2** for a full list of reactions in ‘compartmented CBC model’ and list of abbreviations.

In line with observations from the regression approach, using INCA we obtained a statistically acceptable fit for *C. ohadii* under LL only when considering time points 0s to 20s. For *C. ohadii* under EIL no acceptable fit was obtained for both sets of integrated MID data. Using the mean flux estimates from INCA as well as mean flux estimated from SFCR (**Supplementary Table S4**), we calculated Spearman correlation and found a high qualitative agreement between flux estimates in each algae (*C. reinhardtii* $r_{s}=1,\;p-value<10^{-10}$ , *C. sorokiniana* $r_{s}=0.91,\;p-value<10^{-10}$ , *C. ohadii* $r_{s}=0.89,\;p-value<10^{-10}$ , **Supplementary Table S9**). In contrast to SFCR, INCA was able to match measured starch and sucrose synthesis rates resulting in a two order increase of absolute flux values in comparison to the SFCR approach (**Figure 5**). In addition, this discrepancy resulted in different conclusions that can be drawn from the two approaches. SFCR indicated that the main difference across algae was the sugar produced, i.e. starch in *C. reinhardtii* and *C. sorokiniana* and, in contrast, sucrose in *C. ohadii*; in comparison, the results from INCA showed that, in line with the fitted experimental evidence, starch is the main sugar produced across all algae, with increased production for *C. sorokiniana* and highest for *C. ohadii* (see **Supplementary Table S** **4** for a list of mean flux as well as CI of all model reactions obtained from SFCR and INCA). While the solution obtained from INCA included the correct values for starch and sucrose synthesis rates, it should be also noted that CI obtained from INCA often span several orders of magnitude. In contrast, SFCR was able to predict considerably smaller CIs (**Supplementary Table S4**).

Therefore, we next examined possible reasons for the two order of magnitude difference in flux estimates between INCA and SFCR. The main difference between the modeling with INCA and SFCR is the consideration of pool size as variable or constant (corresponding to mean measurements). Hence, we investigated if the difference of flux estimates may be due to variance in metabolite pool sizes used in INCA and measured pool sizes integrated in the SFCR approach. While the estimated total pool size from INCA matched the measured content for ADPG, RuBP and UDPG, estimates of pool sizes for F6P, FBP, G1P and G6P were different from mean measurements by at least one order of magnitude (**Supplementary Table S7**), underlining that the used metabolic pool size might be the origin of discrepancy in estimated fluxes. Therefore, as a last check, we repeated the estimation of fluxes with SFCR by fixing the pool sizes to those estimated from INCA. This modeling scenario indicated that pool size from INCA cannot explain the differences of two orders of magnitude to estimates from SFCR. In addition, for *C. sorokiniana* we even obtained no acceptable fit to experimental data based on the $\chi^{2}=53812$ . Since no flux towards starch and sucrose synthesis is predicted for *C. sorokiniana* (**Figure 5B**), we also found a decrease in qualitative agreement ( $r_{s}=0.52$ ) between the estimates from SFCR with measured and estimated pool size from INCA (**Supplementary Table S9**). Similarly, also for *C. reinhardtii* no flux towards starch or sucrose synthesis is predicted (**Figure 5A**), although, in contrast to *C. sorokiniana* the $\chi^{2}$ is acceptable ( $\chi^{2}=128.8$ ). The only flux distribution that remains unchanged considering the ordering of fluxes is the one of *C. ohadii* ( $r_{s}=1$ ) (**Figure 5C**). Therefore, pool sizes cannot explain the order difference in estimated flux between INCA and the proposed SFCR approach.

As shown in Section 2.1. the SFCR approach makes use of the Forward Euler approximation to discretize the ODEs. Hence, flux predictions might be affected by large approximation errors as a result of large time steps considered in the experimental setup. To investigate the extent the experimental time steps affect flux predictions from SFCR we used the interpolation of the measured MID data to compare previous results with flux estimation from denser time series (see Methods). We found that the flux estimates obtained from the original time series and the time series with ·t being 1 sec are highly correlated ( r = 0.97, **Supplementary Table S8**) and had no effect on the order of magnitude difference in the estimated fluxes between SFCR and INCA.

## 3 Discussion

The availability of flux estimates for reactions in a metabolic network provides several possible applications. In scenarios in which fluxes are compared between conditions for the same organism, reactions with differential fluxes can be seen as main drivers for the differential behavior for a related trait at a higher level (e.g. growth). Further, in scenarios in which fluxes of the same pathway or network are compared across organisms (e.g. algal species), the reactions with differential fluxes between organisms, can indicate points of intervention to drive the high-level trait towards a desired biotechnological goal. To this end, the fluxes estimated from integration of labeling data can be integrated in genome-scale metabolic networks as constraints in various constraint-based approaches. However, obtaining flux estimates for photosynthetic organisms, particularly in plants, remains challenging due to the large experimental efforts as well as the related computational problems associated with the relevant mathematical formulations.

Here we presented a simple approach, termed SFCR, for flux estimation from nonstationary labeling states in data rich cases. In such data-rich cases, there is no need to simulate MIDs for the considered metabolites, allowing a one-shot robust estimation of fluxes from a single QP formulation. This allows us to speed up the computations at least 100-fold (find best fit for one algae INCA ~7 min vs. ~4 sec in SFCR, considering calculation of CI the speed up is at least 1000-fold, INCA several days vs. ~5 min SFCR) and obtain flux estimates as well as consider multiple modeling scenarios which we discuss here. Interestingly, the SFCR formulation allowed us to investigate compartmentation via a simple sampling approach, significantly reducing the time for parameter estimation. The sampling approach assumed that the ratio between cytosolic and chloroplastic pool is between 0.1 and 0.9. This assumption is in line with predictions from INCA (**Supplementary Table S6**) for *C. reinhardtii* and *C. sorokiniana*; however, it could be further refined by experimental knowledge in later studies. For *C. ohadii*, the compartmentation predicted in INCA indicates larger cytosolic than chloroplastic pools for T3P, FBP and G6P. Note that compartmentation values obtained from INCA correspond to those obtained from the pseudo-reaction added for compartmentation effects. Furthermore, we found differences between the compartmentation ratio from the ratio of estimated pool sizes used in INCA (compare ratios in **Supplementary Table S6 and S7**). Our aim was to then compare the flux estimations from SFCR with those obtained from INCA, as the state-of-the-art approach for flux estimation with data from isotopic nonstationary states in plants (Young et al., 2011; Jazmin et al., 2014; Allen and Young, 2020; Xu et al., 2021; Chu et al., 2022; Xu et al., 2022; Smith et al., 2023). The comparison was meant to point at issues with the assumptions and formulations used by the existing contenders.

While the flux estimates from SFCR seem to match the results from the differential analysis based on the flux distributions from INCA, we observed that the former are two orders of magnitude smaller. Careful investigation suggests that the discrepancies can be due to at least four factors dealing with the treatment of: (1) total pool sizes, (2) sizes of pools actively involved in photosynthetic processes, (3) pool compartmentation, and (4) constraints on boundary fluxes to the modeled system.

Regarding the total pool sizes, we found that usage of the estimates of pool sizes from INCA, for which some metabolites (e.g. F6P and FBP) differ by an order or magnitude, result in no acceptable fit for *C. sorokiniana* and no sugar production for *C. reinhardtii* and *C. sorokiniana* (**Supplementary Table S7, S8**). Thereby, the result shows the importance of precise pool size measurements when integrating them in the approach for flux estimation. In addition, experiments in which we treated pool sizes as free parameters, using compartmentation values from INCA, indicated that no acceptable fit could be achieved for *C. reinhardtii* and that RuBisCO carboxylation rates in *C. ohadii* dropped to values two order of magnitude below those estimated for *C. sorokiniana* (**Supplementary Table S8**).

Another possibility is that only a fraction of the measured pool actively participates in photosynthesis, due to various reasons [e.g. different cell types, microcompartmentation issues (Küken et al., 2018)]. Repeating the analysis with the fixed pool sizes to measurements, with compartmentation from INCA, and rescaling of labelled fraction according to the last time point indicated that the pool size cannot explain the discrepancy in starch and sucrose synthesis flux as well as the difference of two orders of magnitude in absolute flux (**Supplementary Table S8**). The observation that none of the parameter sets taken from INCA (i.e. pool size and compartmentation ratio) resulted in improved agreement between expected flux distribution with respect to order of magnitude and/or sugar synthesis flux indicate that only the interplay of all optimized parameters in INCA (i.e. (compartmented) pool size, compartmentation by pseudo-reactions and isotopomer-specific scaling factor) allows to match the measured starch and sucrose synthesis rates.

Lastly, it is known that boundary fluxes have a large effect on the flux estimates and often lead to numerical issues, which has prompted the usage of local approach that motivated our work. Rescaling the experimental exchange fluxes used by division with factor 100 results in starch and sucrose synthesis rate estimated from SFCR that match the observed pattern, underlining the issue of numerical problems. The rescaling of the exchange flux leads to a good qualitative agreement between flux estimated for individual algae from SFCR and INCA indicated by $r_{S}\geq 0.88$ (**Supplementary Table S9**). Similar agreement was obtained with the original implementation of the SFCR approach ( $r_{S}\geq 0.87$ ) and treating pool sizes as predicted variables ( $r_{S}\geq 0.87$ ). It remains to be investigated how better estimates of the boundary fluxes can be obtained, given the fact that these metabolites are not excreted in the medium, but are integral to the algal cells, unlike other microbial studies (Davidi et al., 2016; Chen and Nielsen, 2021).

Our study showed that in data-rich scenarios, as it is the case for the CBC, as one of the major pathways in photosynthesis, SFCR provides a suitable alternative to INST-MFA approaches (e.g. as implemented in INCA). Recent developments in omics technologies result in high coverage in terms of data for other pathways, including: the TCA cycle and amino acid biosynthesis, and parts of lipid metabolism, for which SFCR can be applied in the future. In addition, the formulation of SFCR could be used to compare the extent to which flux distributions obtained from different constrained-based approaches fit experimentally measured labeling pattern. However, our study also pointed at several issues with flux estimation, even in data rich scenarios, and indicated that future efforts must focus on careful comparison of multiple alternative model structures and provide validations and plausible explanations for the discrepancy between measured and fitted (compartmented) pool sizes. To this end modifications to the approach may need to be introduced, such as rendering it applicable to estimation of fluxes akin to local approaches (Huß and Nikoloski, 2023).

## 4 Methods

### 4.1 Metabolic models

We used two variants of a metabolic model for the CBC differing in their complexity. The first model version, denoted as ‘simple CBC model’, considers only CBC with a branch towards starch synthesis and no compartmentation of metabolite pools. The second model version, denoted as ‘compartmented CBC model’, includes reactions for the CBC and related processes, i.e. photorespiration, starch and sucrose synthesis. The model considers metabolite compartmentation into chloroplast and cytosol. Under the assumptions indicated in the main text, ODEs for the MIDs of RuBP, F6P, FBP, G6P, G1P, and ADPG could be written for the ‘simple CBC model’ and ODEs for the MIDs of RuBP, F6P, FBP, G1P, G6P, UDPG, and ADPG could be written using the ‘compartmented CBC model’. A full list of model reactions and ordinary differential equations can be found in **Supplementary Information 1.1** and **1.2** for the ‘simple CBC model’ and ‘compartmented CBC model’, respectively. Combined with MID measurements for metabolites RuBP, DHAP (used as MID for T3P), F6P, FBP, G1P, G6P, UDPG and ADPG we used the ODEs to find reaction flux distributions that allow to fit measured labeling patterns.

### 4.2 Considered measurement error of MID data for model fit

To assess the quality of the fit we used: (i) the $\chi^{2}$ statistic with degrees of freedom corresponding to the difference of the number of measurements and the number of modelled parameters (corresponding to the rank of stoichiometric matrix N ); (ii) the distribution of residuals over the individual measurements, and assessed whether it follows a normal distribution with a mean of zero and standard deviation of one (**Supplementary Figure S2**). Using the $\chi^{2}$ statistic, the model fit strongly depends on the underlying measurement error. For MID data the standard deviation is often zero for high labeled isotopologues at early time points preventing the model to fit the data. To overcome this problem, we assumed that measurement error, σ , does not depend on time and considered the maximum standard deviation for each isotopologue observed over time as measurement error. In case an isotopologue $M_{m+j}$ had standard deviation of zero across all measured time points, we considered the standard deviation of $M_{m+j-1}$ as measurement error for $M_{m+j}$ in data fitting. The code to obtain the measurement error used in model fit, that was calculated based on the standard deviation of measured MID data, can be found on GitHub (https://github.com/ankueken/MFA_Reg).

### 4.3 Data used in formulation of problems

Having measurements of absolute pool size concentrations and MIDs at five time points 0, 5, 10, 20 and 40s, $t_{i}$ and $\Delta t_{i}$ being {0, 5, 10, 20} and {5, 5, 10, 20}, respectively. Note, that for flux estimation in *C. ohadii* under LL we did not consider the last time point (see main text). Data on metabolite pool size, MID level and starch/sucrose synthesis rates that entered in the program above can be found in **Supplementary Table S2**. To avoid describing differences that only arise due to reactions that carry flux below solver tolerance, we consider reactions with flux below 10^-6^ $nmol\;gDW^{-1}s^{-1}$ to carry no flux. We used B-splines as implemented in Python package Scipy to interpolate the MIDs using each individual replicate.

### 4.4 Bootstrap confidence intervals

To obtain CI and $\chi^{2}$ for the model fit, we used non-parametric bootstrap by running the following procedure: we resampled the residuals ε , obtained from the QP above, with replacement to generate a new set of residuals $\varepsilon^{*}$ . To generate a new bootstrap data set, $\varepsilon^{*}$ is added to the measured MIDs. Then the bootstrap dataset was used as an independent replicate experiment, and the QP was solved to calculate new estimates of model parameters. The procedure was repeated 1000 times and bootstrap model parameters as well as bootstrap $\chi^{2}$ were stored. To obtain 95% CI we computed the 97.5th and the 2.5th percentile of the obtained bootstrap distribution for model parameters and $\chi^{2}$ .

The fit between model prediction and data can be accepted if the obtained $\chi^{2}$ for model fit falls within the interval $\left[\chi_{\frac{\alpha }{2}}^{2}\left(df\right),\;\chi_{1-\frac{\alpha }{2}}^{2}\left(df\right)\right]$ , where α is the significance level, here we use 0.05, and df is the degree of freedom, corresponding to the difference of the number of measurements and the number of modelled parameters (i.e. the rank of the stoichiometric matrix N ) (Young, 2014).

### 4.5 Implementing compartmentation coefficients

For a metabolite present in both the chloroplast [p] and cytosol [c] , the dynamics of the ^13^C labeling of the total pool are the summation of the labeling dynamics observed in the plastid and those observed in the cytosol.


$$Px\left(t_{i}+\text{Δ}t_{i}\right)-Px\left(t_{i}\right)=S^{\left[p\right]}\left(t_{i}\right)v\text{Δ}t_{i}+S^{\left[c\right]}\left(t_{i}\right)v\text{Δ}t_{i}\cdot$$

Hence, in the QP above matrix $\text{S}={\text{S}}^{\left[\text{p}\right]}+{\text{S}}^{\left[\text{c}\right]}$ , with ${\text{S}}^{\left[\text{p}\right]}$ combining MID data for plastidial labeling dynamics and $S^{\left[c\right]}$ combining MID data for cytosolic labeling dynamics. However, compartment specific MID data were not available. Therefore, we substitute MID data in S that model cytosolic labeling dynamics by $M_{m+j}^{\left[c\right]}=c\cdot M_{m+j}^{\left[p\right]}$ , with c denoting the fraction of $\frac{M_{m+j}^{\left[c\right]}}{M_{m+j}^{\left[p\right]}}$ . Assuming that the labeling dynamics of the total pool are predominantly driven by the reactions in the chloroplast, we sampled values for c from the interval [0.1, 0.9]. Since we assume parameters c to show no drastic change for bootstrapped data, we limit the deviation of parameters c chosen during bootstrap sampling to be at most ±2.5% from the parameterization of c used in the best fit.

### 4.6 Flux estimation from SFCR with variable pool sizes

The SFCR approach also allows to treat the pool size parameter P as an unknown variable. This assumption will result in the following change in variance $\sigma^{2}$ entering ${\text{Q}}_{MID}$ in the QP. Assuming that P and $\text{x}\left(t_{i}\right)$ are independent variables the variance of $\text{Y}\left(t_{i}\right)$ is $\sigma_{P}^{2}\cdot \sigma_{x\left(t_{i}\right)}^{2}+\sigma_{P}^{2}{\left(\mu_{x\left(t_{i}\right)}\right)}^{2}+\sigma_{x\left(t_{i}\right)}^{2}{\left(\mu_{P}\right)}^{2}$ . Moreover, the QP solved included $\varepsilon_{P}\in {\mathbb{R}}^{\text{n}\times 1}$ denote the deviation between measured and estimated pool size for metabolites n whose MIDs enter S and $Q_{P}\in {\mathbb{R}}^{n\times n}$ be a diagonal matrix that contains the variance for the measured pool size. Based on these simplifying assumptions, flux estimation with variable pool size corresponds to the following QP in which we minimize chi-square goodness-of-fit measure:


$$\underset{v,\varepsilon_{MID},\varepsilon_{V},\varepsilon_{P}}{\text{min }}\varepsilon_{MID}\cdot Q_{MID}\cdot \varepsilon_{MID}{}^{T}+\varepsilon_{P}\cdot Q_{P}\cdot \epsilon_{P}{}^{T}+\varepsilon_{V}\cdot Q_{V}\cdot \varepsilon_{V}{}^{T}$$

s.t.

$$S\left(t_{i}\right)v\Delta t_{i}+\varepsilon_{MID}\left(t_{i}\right)=Px\left(t_{i}+\Delta t_{i}\right)-Px\left(t_{i}\right)$$

Nv=0

$$v_{exc}+\varepsilon_{V}=v_{exc,measured}$$

$$P+\varepsilon_{P}=P_{measured}$$

$$v_{min}1\leq v\leq v_{max}1$$

$$-10^{6}1\leq \varepsilon_{MID}\leq 10^{6}1$$

$$-10^{6}1\leq \varepsilon_{P}\leq 10^{6}1\;\;\;\;\;,$$

$$-10^{6}1\leq \varepsilon_{V}\leq 10^{6}1$$

## Data availability statement

Publicly available datasets were analyzed in this study. This data can be found here: https://github.com/ankueken/MFA_Reg.

## Author contributions

Conceptualization, ZN; Experimental data, HT; Implementation, AK; Formal Analysis, AK, ZN; Writing – Original Draft, AK, ZN; Writing – Review and Editing, AK, HT, and ZN. All authors contributed to the article and approved the submitted version.
