# Supplementary material for: A simulation-free constrained regression approach for flux estimation in isotopically nonstationary metabolic flux analysis with applications in microalgae
Source: Front Plant Sci. 2023 Nov 23;14:1140829. doi: 10.3389/fpls.2023.1140829 (PMC10702240; doi:10.3389/fpls.2023.1140829)
Supplement: Supplementary file 1 [file DataSheet_1.docx]

Supplementary Material

A simulation-free constrained regression approach for flux estimation in istotopically nonstationary metabolic flux analysis with applications in microalgae

**Anika Küken, Haim Treves, Zoran Nikoloski^*^**

*** Correspondence:** Zoran Nikoloski: nikoloski@mpimp-golm.mpg.de

# Supplementary Information

## Simple CBC model

### Model reactions

| Reaction name | Reaction formula |
| --- | --- |
| $\mathbf{T}_{\boldsymbol{CO}\boldsymbol{2}}$ | $\to{CO}_{2}$ |
| $\boldsymbol{RUBISCO}_{\boldsymbol{CO}\boldsymbol{2}}$ | $RUBP+ {CO}_{2}\to2\cdot3PGA$ |
| $\boldsymbol{RUBISCO}_{\boldsymbol{O}\boldsymbol{2}}$ | $RUBP\to3PGA$ |
| $\boldsymbol{GAPDH}$ | $3PGA\to T3P$ |
| $\boldsymbol{FBA}$ | $2\cdot T3P\leftrightarrow FBP$ |
| $\boldsymbol{PFP}$ | $FBP\to F6P$ |
| $\boldsymbol{TK}\boldsymbol{3}$ | $F6P\leftrightarrow E4P+EC2$ |
| $\boldsymbol{ALD}$ | $T3P+E4P\to SBP$ |
| $\boldsymbol{SBPase}$ | $SBP\to S7P$ |
| $\boldsymbol{TK}\boldsymbol{1}$ | $T3P+EC2\leftrightarrow PP$ |
| $\boldsymbol{TK}\boldsymbol{2}$ | $S7P\leftrightarrow R5P+EC2$ |
| $\boldsymbol{PPI}$ | $R5P\leftrightarrow PP$ |
| $\boldsymbol{PRK}$ | $PP\to RUBP$ |
| $\boldsymbol{PGI}$ | $F6P\leftrightarrow G6P$ |
| $\boldsymbol{PGM}$ | $G6P\leftrightarrow G1P$ |
| $\boldsymbol{AGP}$ | $G1P\to ADPG$ |
| $\boldsymbol{SS}$ | $ADPG\to$ |

### Abbreviations

Reactions / Enzymes: AGP - Glucose-1-phosphate adenylyltransferase; ALD - Fructose-bisphosphate aldolase; GAPDH – lumped reaction of Phosphoglycerate kinase, glyceraldehyde-3-phosphate dehydrogenase and Triose-phosphate isomerase; FBA - Fructose-1,6-phosphate aldolase; PFP - Fructose 1,6-bisphosphatase; PGI - Glucose-6-phosphate isomerase; PGM - Phosphoglucomutase; PPI - Ribose-5-phosphate isomerase; PRK - Phosphoribulokinase; RUBISCO_CO2_ – Ribulose-1,5-bisphosphate carboxylase; RUBISCO_O2_ – Ribulose-1,5-bisphosphate oxygenase; SBPase - Sedulose-1,7-bisphosphatase; SS - Starch synthase; T_CO2_ – CO_2_ import

Metabolites: 3PGA - 3-phosphoglycerate; ADPG - ADP-glucose; CO_2_ - carbon dioxide; E4P - erythrose-4-phosphate; EC2 - enzyme-bound carbon fragment; F6P - fructose-6-phosphate; FBP - fructose-1,6-bisphosphatase; G1P - glucose-1-phosphate; G6P - glucose-6-phosphate; PP - pentose-5-phosphate; PP – pentose-5-phosphates; R5P - ribose-5-phosphate; RUBP - ribulose-1,5-bisophosphate; S7P - sedoeheptulose-7-phosphate; SBP - sedoheptulose-1,7-bisophosphate; T3P - triose-phosphate

### Differential equations for metabolite MIDs

When the differential equation applies to all MIDs of metabolite $M$ we use the general description of $M_{m+x}$, where $x$ is an integer value indicating the number of labeled C atoms.

$\frac{\boldsymbol{dRuBP}}{\boldsymbol{dt}}\boldsymbol{=}\boldsymbol{v}_{\boldsymbol{PRK}}\boldsymbol{-(}\boldsymbol{v}_{\boldsymbol{RUBISC}\boldsymbol{O}_{\boldsymbol{CO}\boldsymbol{2}}}\boldsymbol{+}\boldsymbol{v}_{\boldsymbol{RUBISC}\boldsymbol{O}_{\boldsymbol{O}\boldsymbol{2}}}\boldsymbol{)}$

${P_{RuBP}(RuBP}_{m+x}\left( t_{i}+\Delta t_{i} \right)-RuBP_{m+x}\left( t_{i} \right))=v_{PRK}\cdot{PP}_{m+x}\left( t_{i} \right)\Delta t_{i}-(v_{RUBISCO_{CO2}}+v_{RUBISCO_{O2}})\cdot{RuBP}_{m+x}\left( t_{i} \right)\Delta t_{i}$

$\frac{\boldsymbol{dF}\boldsymbol{6}\boldsymbol{P}}{\boldsymbol{dt}}\boldsymbol{=}\boldsymbol{v}_{\boldsymbol{PFP}}\boldsymbol{+}\boldsymbol{v}_{\boldsymbol{PGI.rev}}\boldsymbol{-(}\boldsymbol{v}_{\boldsymbol{TK}\boldsymbol{3}}\boldsymbol{+}\boldsymbol{v}_{\boldsymbol{PGI}}\boldsymbol{)}$

$P_{F6P}\left( {F6P}_{m+x}\left( t_{i}+\Delta t_{i} \right)-F6P_{m+x}\left( t_{i} \right) \right)=v_{PFP}\cdot{FBP}_{m+x}\left( t_{i} \right)\Delta t_{i}+v_{PGI.rev}\cdot{G6P}_{m+x}\left( t_{i} \right)\Delta t_{i}-\left( v_{TK3}+v_{PGI} \right)\cdot{F6P}_{m+x}\left( t_{i} \right)\Delta t_{i}$

$\frac{\boldsymbol{dFBP}}{\boldsymbol{dt}}\boldsymbol{=}\boldsymbol{v}_{\boldsymbol{PFP.rev}}\boldsymbol{+}\boldsymbol{v}_{\boldsymbol{FBA}}\boldsymbol{-(}\boldsymbol{v}_{\boldsymbol{PFP}}\boldsymbol{+}\boldsymbol{v}_{\boldsymbol{FBA.rev}}\boldsymbol{)}$

$$P_{FBP}({FBP}_{m+0}\left( t_{i}+\Delta t_{i} \right)-FBP_{m+0}\left( t_{i} \right))=v_{FBA}\cdot{T3P}_{m+0}\left( t_{i} \right)\cdot{T3P}_{m+0}\left( t_{i} \right)\Delta t_{i}+v_{PFP.rev}\cdot{F6P}_{m+0}\left( t_{i} \right)\Delta t_{i}-\left( v_{PFP}+v_{FBA.rev} \right)\cdot{FBP}_{m+0}\left( t_{i} \right)\Delta t_{i}$$

$$P_{FBP}({FBP}_{m+1}\left( t_{i}+\Delta t_{i} \right)-FBP_{m+1}\left( t_{i} \right))=v_{FBA}\cdot{T3P}_{m+1}\left( t_{i} \right)\cdot{T3P}_{m+0}\left( t_{i} \right)\Delta t_{i}+v_{PFP.rev}\cdot{F6P}_{m+1}\left( t_{i} \right)\Delta t_{i}-\left( v_{PFP}+v_{FBA.rev} \right)\cdot{FBP}_{m+1}\left( t_{i} \right)\Delta t_{i}$$

$$P_{FBP}({FBP}_{m+2}\left( t_{i}+\Delta t_{i} \right)-FBP_{m+2}\left( t_{i} \right))=v_{FBA}\cdot\left( {T3P}_{m+2}\left( t_{i} \right)\cdot{T3P}_{m+0}\left( t_{i} \right)+{T3P}_{m+1}\left( t_{i} \right)\cdot{T3P}_{m+1}\left( t_{i} \right) \right)\Delta t_{i}+v_{PFP.rev}\cdot{F6P}_{m+2}\left( t_{i} \right)\Delta t_{i}-\left( v_{PFP}+v_{FBA.rev} \right)\cdot{FBP}_{m+2}\left( t_{i} \right)\Delta t_{i}$$

$$P_{FBP}({FBP}_{m+3}\left( t_{i}+\Delta t_{i} \right)-FBP_{m+3}\left( t_{i} \right))=v_{FBA}\cdot\left( {T3P}_{m+3}\left( t_{i} \right)\cdot{T3P}_{m+0}\left( t_{i} \right)+{T3P}_{m+2}\left( t_{i} \right)\cdot{T3P}_{m+1}\left( t_{i} \right) \right)\Delta t_{i}+v_{PFP.rev}\cdot{F6P}_{m+3}\left( t_{i} \right)\Delta t_{i}-\left( v_{PFP}+v_{FBA.rev} \right)\cdot{FBP}_{m+3}\left( t_{i} \right)\Delta t_{i}$$

$$P_{FBP}({FBP}_{m+4}\left( t_{i}+\Delta t_{i} \right)-FBP_{m+4}\left( t_{i} \right))=v_{FBA}\cdot\left( {T3P}_{m+3}\left( t_{i} \right)\cdot{T3P}_{m+1}\left( t_{i} \right)+{T3P}_{m+2}\left( t_{i} \right)\cdot{T3P}_{m+2}\left( t_{i} \right) \right)\Delta t_{i}+v_{PFP.rev}\cdot{F6P}_{m+4}\left( t_{i} \right)\Delta t_{i}-\left( v_{PFP}+v_{FBA.rev} \right)\cdot{FBP}_{m+4}\left( t_{i} \right)\Delta t_{i}$$

$$P_{FBP}({FBP}_{m+5}\left( t_{i}+\Delta t_{i} \right)-FBP_{m+5}\left( t_{i} \right))=v_{FBA}\cdot{T3P}_{m+3}\left( t_{i} \right)\cdot{T3P}_{m+2}\left( t_{i} \right)\Delta t_{i}+v_{PFP.rev}\cdot{F6P}_{m+5}\left( t_{i} \right)\Delta t_{i}-\left( v_{PFP}+v_{FBA.rev} \right)\cdot{FBP}_{m+5}\left( t_{i} \right)\Delta t_{i}$$

$${P_{FBP}(FBP}_{m+6}\left( t_{i}+\Delta t_{i} \right)-FBP_{m+6}\left( t_{i} \right))=v_{FBA}\cdot{T3P}_{m+3}\left( t_{i} \right)\cdot{T3P}_{m+3}\left( t_{i} \right)\Delta t_{i}+v_{PFP.rev}\cdot{F6P}_{m+6}\left( t_{i} \right)\Delta t_{i}-\left( v_{PFP}+v_{FBA.rev} \right)\cdot{FBP}_{m+6}\left( t_{i} \right)\Delta t_{i}$$

$\frac{\boldsymbol{dG}\boldsymbol{6}\boldsymbol{P}}{\boldsymbol{dt}}\boldsymbol{=}\boldsymbol{v}_{\boldsymbol{PGI}}\boldsymbol{+}\boldsymbol{v}_{\boldsymbol{PGM.rev}}\boldsymbol{-(}\boldsymbol{v}_{\boldsymbol{PGM}}\boldsymbol{+}\boldsymbol{v}_{\boldsymbol{PGI.rev}}\boldsymbol{)}$

$P_{G6P}\left( {G6P}_{m+x}\left( t_{i}+\Delta t_{i} \right)-G6P_{m+x}\left( t_{i} \right) \right)=v_{PGI}\cdot{F6P}_{m+x}\left( t_{i} \right)\Delta t_{i}+v_{PGM.rev}\cdot{G1P}_{m+x}\left( t_{i} \right)\Delta t_{i}-\left( v_{PGM}+v_{PGI.rev} \right)\cdot{G6P}_{m+x}\left( t_{i} \right)\Delta t_{i}$

$\frac{\boldsymbol{dG}\boldsymbol{1}\boldsymbol{P}}{\boldsymbol{dt}}\boldsymbol{=}\boldsymbol{v}_{\boldsymbol{PGM}}\boldsymbol{-(}\boldsymbol{v}_{\boldsymbol{AGP}}\boldsymbol{+}\boldsymbol{v}_{\boldsymbol{PGM.rev}}\boldsymbol{)}$

$$P_{G1P}({G1P}_{m+x}\left( t_{i}+\Delta t_{i} \right)-G1P_{m+x}\left( t_{i} \right))=v_{PGM}\cdot{G6P}_{m+x}\left( t_{i} \right)\Delta t_{i}-\left( v_{AGP}+v_{PGM.rev} \right)\cdot{G1P}_{m+x}\left( t_{i} \right)\Delta t_{i}$$

$\frac{\boldsymbol{dADPG}}{\boldsymbol{dt}}\boldsymbol{=}\boldsymbol{v}_{\boldsymbol{AGP}}\boldsymbol{-}\boldsymbol{v}_{\boldsymbol{SS}}$

$$P_{G1P}({G1P}_{m+x}\left( t_{i}+\Delta t_{i} \right)-G1P_{m+x}\left( t_{i} \right))=v_{AGP}\cdot{G1P}_{m+x}\left( t_{i} \right)\Delta t_{i}-v_{SS}\cdot{ADPG}_{m+x}\left( t_{i} \right)\Delta t_{i}$$

## Compartmented CBC model

### Model reactions

| Reaction name | Reaction formula |
| --- | --- |
| Calvin-Benson-Basham cycle | |
| $\mathbf{T}_{\boldsymbol{CO}\boldsymbol{2}}$ | $\to{CO}_{2}$ |
| $\boldsymbol{RUBISCO}_{\boldsymbol{CO}\boldsymbol{2}}$ | $RUBP+ {CO}_{2}\to2\cdot3PGA.p$ |
| $\boldsymbol{RUBISCO}_{\boldsymbol{O}\boldsymbol{2}}$ | $RUBP\to3PGA.p+2PG$ |
| $\boldsymbol{GAPDH}$ | $3PGA.p\to T3P.p$ |
| $\boldsymbol{FBA}\boldsymbol{.}\boldsymbol{p}$ | $2\cdot T3P.p\leftrightarrow FBP.p$ |
| $\boldsymbol{PFP.p}$ | $FBP.p\to F6P.p$ |
| $\boldsymbol{TK}\boldsymbol{3}$ | $F6P.p\leftrightarrow E4P+EC2$ |
| $\boldsymbol{ALD}$ | $T3P.p+E4P\to SBP$ |
| $\boldsymbol{SBPase}$ | $SBP\to S7P$ |
| $\boldsymbol{TK}\boldsymbol{1}$ | $T3P.p+EC2\leftrightarrow PP$ |
| $\boldsymbol{TK}\boldsymbol{2}$ | $S7P\leftrightarrow R5P+EC2$ |
| $\boldsymbol{PPI}$ | $R5P\leftrightarrow PP$ |
| $\boldsymbol{PRK}$ | $PP\to RUBP$ |
| Photorespiration | |
| $\boldsymbol{PGP}$ | $2PG\to GLY$ |
| $\boldsymbol{GDC}$ | $2\cdot GLY\to SER+ {CO}_{2}$ |
| $\boldsymbol{SGA}\boldsymbol{1}$ | $SER\to GA$ |
| $\boldsymbol{GK}$ | $GA\leftrightarrow3PGA$ |
| Starch synthesis | |
| $\boldsymbol{PGI.p}$ | $F6P.p\leftrightarrow G6P.p$ |
| $\boldsymbol{PGM.p}$ | $G6P.p\leftrightarrow G1P.p$ |
| $\boldsymbol{AGP}$ | $G1P.p\to ADPG$ |
| $\boldsymbol{SS}$ | $ADPG\to$ |
| Sucrose synthesis | |
| $\boldsymbol{FBA}\boldsymbol{.c}$ | $2\cdot T3P.c\leftrightarrow FBP.c$ |
| $\boldsymbol{PFP.c}$ | $FBP.c\to F6P.c$ |
| $\boldsymbol{PGI.c}$ | $F6P.c\leftrightarrow G6P.c$ |
| $\boldsymbol{PGM.c}$ | $G6P.c\leftrightarrow G1P.c$ |
| $\boldsymbol{GPU}$ | $G1P.c\to UDPG$ |
| $\boldsymbol{SPS}$ | $UDPG+F6P.c\to$ |
| Branch towards TCA cycle | |
| $\boldsymbol{PGAM.c}$ | $3PGA.c\to PEP.c$ |
| $\boldsymbol{PE.c}$ | $PEP.c\to$ |
| Branch towards fatty acid synthesis / shikimate pathway | |
| $\boldsymbol{PGAM.p}$ | $3PGA.p\to PEP.p$ |
| $\boldsymbol{PE.p}$ | $PEP.p\to$ |
| Transport plastid - cytosol | |
| $\mathbf{T}_{\boldsymbol{3}\boldsymbol{PGA}}$ | $3PGA.p\leftrightarrow3PGA.c$ |
| $\mathbf{T}_{\boldsymbol{T}\boldsymbol{3}\boldsymbol{P}}$ | $T3P.p\leftrightarrow T3P.c$ |
| $\mathbf{T}_{\boldsymbol{PEP}}$ | $PEP.p\leftrightarrow PEP.c$ |

### Abbreviations

Compartments: .p – chloroplast; .c – cytosol

Reactions / Enzymes: AGP - Glucose-1-phosphate adenylyltransferase; ALD - Fructose-bisphosphate aldolase; GAPDH – lumped reaction of Phosphoglycerate kinase, glyceraldehyde-3-phosphate dehydrogenase and Triose-phosphate isomerase; GDC – glycine dehydrogenase complex; GK - Glycerate kinase; GPU - Glucose-1-phosphate uridylyltransferase; FBA - Fructose-1,6-phosphate aldolase; PE – PEP export; PFP - Fructose 1,6-bisphosphatase; PGAM - Phosphoglyceratmutase; PGI - Glucose-6-phosphate isomerase; PGM - Phosphoglucomutase; PGP - Phosphoglycolate phosphatase; PPI - Ribose-5-phosphate isomerase; PRK - Phosphoribulokinase; RUBISCO_CO2_ – Ribulose-1,5-bisphosphate carboxylase; RUBISCO_O2_ – Ribulose-1,5-bisphosphate oxygenase; SBPase - Sedulose-1,7-bisphosphatase; SGA1 – Serine-glyoxylate transaminase; SS - Starch synthase; SPS - Sucrose-phosphate synthase; T_3PGA_ – transport 3PGA; T_CO2_ – CO_2_ import; TK1/2/3 – Transketolase; T_PEP_ - transport PEP; T_T3P_ – transport T3P

Metabolites: 2PG - 2-phosphoglycolate; 3PGA - 3-phosphoglycerate; ADPG - ADP-glucose; CO_2_ - carbon dioxide; E4P - erythrose-4-phosphate; EC2 - enzyme-bound carbon fragment; F6P - fructose-6-phosphate; FBP - fructose-1,6-bisphosphatase; G1P - glucose-1-phosphate; G6P - glucose-6-phosphate; GA – glycerate; GLY – glycine; PP - pentose-5-phosphate; PEP – phosphoenolpyruvate; PP – pentose-5-phosphates; R5P - ribose-5-phosphate; RUBP - ribulose-1,5-bisophosphate; S7P - sedoeheptulose-7-phosphate; SBP - sedoheptulose-1,7-bisophosphate; SER – serine; T3P - triose-phosphate; UDPG - UDP-glucose

### Differential equations for metabolite MIDs

Using the model of CBC, photorespiration, starch and sucrose synthesis above we can write ordinary differential equations for the MIDs of RuBP, F6P, FBP, G1P, G6P, UDPG and ADPG. When the differential equation applies to all MIDs of metabolite $M$ we use the general description of $M_{m+x}$, where $x$ is an integer value indicating the number of labeled C atoms. To implement compartmentation, cytosolic MIDs are represented in terms of plastidial MIDs using coefficients $\{c,c^{I},c^{II},c^{III},c^{IV}\}$ being fractions of cytosolic and plastidial metabolite MID for metabolites T3P, F6P, FBP, G6P and G1P, respectively (also see section ‘Implementing compartmentation coefficients’ in Methods main text). Since the system is in a metabolic steady state, we assume that coefficients $\{c,c^{I},c^{II},c^{III},c^{IV}\}$ do not depend on time.

$\frac{\boldsymbol{dRuBP.p}}{\boldsymbol{dt}}\boldsymbol{=}\boldsymbol{v}_{\boldsymbol{PRK}}\boldsymbol{-(}\boldsymbol{v}_{\boldsymbol{RUBISC}\boldsymbol{O}_{\boldsymbol{CO}\boldsymbol{2}}}\boldsymbol{+}\boldsymbol{v}_{\boldsymbol{RUBISC}\boldsymbol{O}_{\boldsymbol{O}\boldsymbol{2}}}\boldsymbol{)}$

${P_{RuBP}(RuBP}_{m+x}\left( t_{i}+\Delta t_{i} \right)-RuBP_{m+x}\left( t_{i} \right))=v_{PRK}\cdot{PP}_{m+x}\left( t_{i} \right)\Delta t_{i}-(v_{RUBISCO_{CO2}}+v_{RUBISCO_{O2}})\cdot{RuBP}_{m+x}\left( t_{i} \right)\Delta t_{i}$

$\frac{\boldsymbol{dFBP}}{\boldsymbol{dt}}\boldsymbol{=}\boldsymbol{v}_{\boldsymbol{PFP.p.rev}}\boldsymbol{+}\boldsymbol{v}_{\boldsymbol{PFP.c.rev}}\boldsymbol{+}\boldsymbol{v}_{\boldsymbol{FBA.p}}\boldsymbol{+}\boldsymbol{v}_{\boldsymbol{FBA.c}}\boldsymbol{-(}\boldsymbol{v}_{\boldsymbol{PFP.p}}\boldsymbol{+}\boldsymbol{v}_{\boldsymbol{PFP.c}}\boldsymbol{+}\boldsymbol{v}_{\boldsymbol{FBA.p.rev}}\boldsymbol{+}\boldsymbol{v}_{\boldsymbol{FBA.c.rev}}\boldsymbol{)}$

$$P_{FBP}\left( {FBP}_{m+0}\left( t_{i}+\Delta t_{i} \right)-FBP_{m+0}\left( t_{i} \right) \right)=v_{FBA.p}\cdot{T3P}_{m+0}\left( t_{i} \right)\cdot{T3P}_{m+0}\left( t_{i} \right)\Delta t_{i}+v_{PFP.p.rev}\cdot{F6P}_{m+0}\left( t_{i} \right)\Delta t_{i}+v_{FBA.c}\cdot c\cdot{T3P}_{m+0}\left( t_{i} \right)\cdot c\cdot{T3P}_{m+0}\left( t_{i} \right)\Delta t_{i}+v_{PFP.c.rev}\cdot{c^{I}\cdot F6P}_{m+0}\left( t_{i} \right)\Delta t_{i}-\left( v_{PFP.p}+v_{FBA.p.rev} \right)\cdot{FBP}_{m+0}\left( t_{i} \right)\Delta t_{i}-\left( v_{PFP.c}+v_{FBA.c.rev} \right)\cdot{c^{II}\cdot FBP}_{m+0}\left( t_{i} \right)\Delta t_{i}$$

$$P_{FBP}\left( {FBP}_{m+1}\left( t_{i}+\Delta t_{i} \right)-FBP_{m+1}\left( t_{i} \right) \right)=v_{FBA.p}\cdot{T3P}_{m+1}\left( t_{i} \right)\cdot{T3P}_{m+0}\left( t_{i} \right)\Delta t_{i}+v_{PFP.p.rev}\cdot{F6P}_{m+1}\left( t_{i} \right)\Delta t_{i}+v_{FBA.c}\cdot c\cdot{T3P}_{m+1}\left( t_{i} \right)\cdot{c\cdot T3P}_{m+0}\left( t_{i} \right)\Delta t_{i}+v_{PFP.c.rev}\cdot c^{I}\cdot{F6P}_{m+1}\left( t_{i} \right)\Delta t_{i}-\left( v_{PFP.p}+v_{FBA.p.rev} \right)\cdot{FBP}_{m+1}\left( t_{i} \right)\Delta t_{i}-\left( v_{PFP.c}+v_{FBA.c.rev} \right)\cdot{c^{II}\cdot FBP}_{m+1}\left( t_{i} \right)\Delta t_{i}$$

$$P_{FBP}\left( {FBP}_{m+2}\left( t_{i}+\Delta t_{i} \right)-FBP_{m+2}\left( t_{i} \right) \right)=v_{FBA.p}\cdot\left( {T3P}_{m+2}\left( t_{i} \right)\cdot{T3P}_{m+0}\left( t_{i} \right)+{T3P}_{m+1}\left( t_{i} \right)\cdot{T3P}_{m+1}\left( t_{i} \right) \right)\Delta t_{i}+v_{PFP.p.rev}\cdot{F6P}_{m+2}\left( t_{i} \right)\Delta t_{i}+v_{FBA.c}\cdot\left( c\cdot{T3P}_{m+2}\left( t_{i} \right)\cdot c\cdot{T3P}_{m+0}\left( t_{i} \right)+{c\cdot T3P}_{m+1}\left( t_{i} \right)\cdot c\cdot{T3P}_{m+1}\left( t_{i} \right) \right)\Delta t_{i}+v_{PFP.c.rev}\cdot{c^{I}\cdot F6P}_{m+2}\left( t_{i} \right)\Delta t_{i}-\left( v_{PFP.p}+v_{FBA.p.rev} \right)\cdot{FBP}_{m+2}\left( t_{i} \right)\Delta t_{i}-\left( v_{PFP.c}+v_{FBA.c.rev} \right)\cdot{c^{II}\cdot FBP}_{m+2}\left( t_{i} \right)\Delta t_{i}$$

$$P_{FBP}\left( {FBP}_{m+3}\left( t_{i}+\Delta t_{i} \right)-FBP_{m+3}\left( t_{i} \right) \right)=v_{FBA.p}\cdot\left( {T3P}_{m+3}\left( t_{i} \right)\cdot{T3P}_{m+0}\left( t_{i} \right)+{T3P}_{m+2}\left( t_{i} \right)\cdot{T3P}_{m+1}\left( t_{i} \right) \right)\Delta t_{i}+v_{PFP.p.rev}\cdot{F6P}_{m+3}\left( t_{i} \right)\Delta t_{i}+v_{FBA.c}\cdot\left( {c\cdot T3P}_{m+3}\left( t_{i} \right)\cdot c\cdot{T3P}_{m+0}\left( t_{i} \right)+{c\cdot T3P}_{m+2}\left( t_{i} \right)\cdot{c\cdot T3P}_{m+1}\left( t_{i} \right) \right)\Delta t_{i}+v_{PFP.c.rev}\cdot{c^{I}\cdot F6P}_{m+3}\left( t_{i} \right)\Delta t_{i}-\left( v_{PFP.p}+v_{FBA.p.rev} \right)\cdot{FBP}_{m+3}\left( t_{i} \right)\Delta t_{i}-\left( v_{PFP.c}+v_{FBA.c.rev} \right)\cdot c^{II}\cdot{FBP}_{m+3}\left( t_{i} \right)\Delta t_{i}$$

$$P_{FBP}\left( {FBP}_{m+4}\left( t_{i}+\Delta t_{i} \right)-FBP_{m+4}\left( t_{i} \right) \right)=v_{FBA.p}\cdot\left( {T3P}_{m+3}\left( t_{i} \right)\cdot{T3P}_{m+1}\left( t_{i} \right)+{T3P}_{m+2}\left( t_{i} \right)\cdot{T3P}_{m+2}\left( t_{i} \right) \right)\Delta t_{i}+v_{PFP.p.rev}\cdot{F6P}_{m+4}\left( t_{i} \right)\Delta t_{i}+v_{FBA.c}\cdot\left( c\cdot{T3P}_{m+3}\left( t_{i} \right)\cdot c\cdot{T3P}_{m+1}\left( t_{i} \right)+{c\cdot T3P}_{m+2}\left( t_{i} \right)\cdot{c\cdot T3P}_{m+2}\left( t_{i} \right) \right)\Delta t_{i}+v_{PFP.c.rev}\cdot{c^{I}\cdot F6P}_{m+4}\left( t_{i} \right)\Delta t_{i}-\left( v_{PFP.p}+v_{FBA.p.rev} \right)\cdot{FBP}_{m+4}\left( t_{i} \right)\Delta t_{i}-\left( v_{PFP.c}+v_{FBA.c.rev} \right)\cdot{c^{II}\cdot FBP}_{m+4}\left( t_{i} \right)\Delta t_{i}$$

$$P_{FBP}\left( {FBP}_{m+5}\left( t_{i}+\Delta t_{i} \right)-FBP_{m+5}\left( t_{i} \right) \right)=v_{FBA.p}\cdot{T3P}_{m+3}\left( t_{i} \right)\cdot{T3P}_{m+2}\left( t_{i} \right)\Delta t_{i}+v_{PFP.p.rev}\cdot{F6P}_{m+5}\left( t_{i} \right)\Delta t_{i}+v_{FBA.c}\cdot c\cdot{T3P}_{m+3}\left( t_{i} \right)\cdot c\cdot{T3P}_{m+2}\left( t_{i} \right)\Delta t_{i}+v_{PFP.c.rev}\cdot c^{I}\cdot{F6P}_{m+5}\left( t_{i} \right)\Delta t_{i}-\left( v_{PFP.p}+v_{FBA.p.rev} \right)\cdot{FBP}_{m+5}\left( t_{i} \right)\Delta t_{i}-\left( v_{PFP.c}+v_{FBA.c.rev} \right)\cdot{c^{II}\cdot FBP}_{m+5}\left( t_{i} \right)\Delta t_{i}$$

$${P_{FBP}(FBP}_{m+6}\left( t_{i}+\Delta t_{i} \right)-FBP_{m+6}\left( t_{i} \right))=v_{FBA.p}\cdot{T3P}_{m+3}\left( t_{i} \right)\cdot{T3P}_{m+3}\left( t_{i} \right)\Delta t_{i}+v_{PFP.p.rev}\cdot{F6P}_{m+6}\left( t_{i} \right)\Delta t_{i}+v_{FBA.c}\cdot c\cdot{T3P}_{m+3}\left( t_{i} \right)\cdot c\cdot{T3P}_{m+3}\left( t_{i} \right)\Delta t_{i}+v_{PFP.c.rev}\cdot c^{I}\cdot{F6P}_{m+6}\left( t_{i} \right)\Delta t_{i}-\left( v_{PFP.p}+v_{FBA.p.rev} \right)\cdot{FBP}_{m+6}\left( t_{i} \right)\Delta t_{i}-\left( v_{PFP.c}+v_{FBA.c.rev} \right)\cdot c^{II}\cdot{FBP}_{m+6}\left( t_{i} \right)\Delta t_{i}$$

$\frac{\boldsymbol{dF}\boldsymbol{6}\boldsymbol{P}}{\boldsymbol{dt}}\boldsymbol{=}\boldsymbol{v}_{\boldsymbol{PFP.p}}\boldsymbol{+}\boldsymbol{v}_{\boldsymbol{PFP.c}}\boldsymbol{+}\boldsymbol{v}_{\boldsymbol{PGI.p.rev}}\boldsymbol{+}\boldsymbol{v}_{\boldsymbol{PGI.c.rev}}\boldsymbol{-(}\boldsymbol{v}_{\boldsymbol{TK}\boldsymbol{3}}\boldsymbol{+}\boldsymbol{v}_{\boldsymbol{PGI.p}}\boldsymbol{+}\boldsymbol{v}_{\boldsymbol{PGI.c}}\boldsymbol{+}\boldsymbol{v}_{\boldsymbol{SPS}}\boldsymbol{)}$

$$P_{F6P}\left( {F6P}_{m+x}\left( t_{i}+\Delta t_{i} \right)-F6P_{m+x}\left( t_{i} \right) \right)=v_{PFP.p}\cdot{FBP}_{m+x}\left( t_{i} \right)\Delta t_{i}+v_{PFP.c}\cdot c^{II}\cdot{FBP}_{m+x}\left( t_{i} \right)\Delta t_{i}+v_{PGI.p.rev}\cdot{G6P}_{m+x}\left( t_{i} \right)\Delta t_{i}+v_{PGI.c.rev}\cdot c^{III}\cdot{G6P}_{m+x}\left( t_{i} \right)\Delta t_{i}-\left( v_{TK3}+v_{PGI.p} \right)\cdot{F6P}_{m+x}\left( t_{i} \right)\Delta t_{i}-\left( v_{PGI.c}+v_{SPS} \right)\cdot c^{I}\cdot{F6P}_{m+x}\left( t_{i} \right)\Delta t_{i}$$

$\frac{\boldsymbol{dG}\boldsymbol{1}\boldsymbol{P}}{\boldsymbol{dt}}\boldsymbol{=}\boldsymbol{v}_{\boldsymbol{PGM.p}}\boldsymbol{+}\boldsymbol{v}_{\boldsymbol{PGM.c}}\boldsymbol{-(}\boldsymbol{v}_{\boldsymbol{AGP}}\boldsymbol{+}\boldsymbol{v}_{\boldsymbol{PGU}}\boldsymbol{+}\boldsymbol{v}_{\boldsymbol{PGM.p.rev}}\boldsymbol{+}\boldsymbol{v}_{\boldsymbol{PGM.c.rev}}\boldsymbol{)}$

$$P_{G1P}\left( {G1P}_{m+x}\left( t_{i}+\Delta t_{i} \right)-G1P_{m+x}\left( t_{i} \right) \right)=v_{PGM.p}\cdot{G6P}_{m+x}\left( t_{i} \right)\Delta t_{i}+v_{PGM.c}\cdot c^{III}\cdot{G6P}_{m+x}\left( t_{i} \right)\Delta t_{i}-\left( v_{AGP}+v_{PGM.p.rev} \right)\cdot{G1P}_{m+x}\left( t_{i} \right)\Delta t_{i}-\left( v_{PGU}+v_{PGM.c.rev} \right)\cdot c^{IV}\cdot{G1P}_{m+x}\left( t_{i} \right)\Delta t_{i}$$

$\frac{\boldsymbol{dG}\boldsymbol{6}\boldsymbol{P}}{\boldsymbol{dt}}\boldsymbol{=}\boldsymbol{v}_{\boldsymbol{PGI.p}}\boldsymbol{+}\boldsymbol{v}_{\boldsymbol{PGI.c}}\boldsymbol{+}\boldsymbol{v}_{\boldsymbol{PGM.p.rev}}\boldsymbol{+}\boldsymbol{v}_{\boldsymbol{PGM.c.rev}}\boldsymbol{-(}\boldsymbol{v}_{\boldsymbol{PGM.p}}\boldsymbol{+}\boldsymbol{v}_{\boldsymbol{PGM.c}}\boldsymbol{+}\boldsymbol{v}_{\boldsymbol{PGI.p.rev}}\boldsymbol{+}\boldsymbol{v}_{\boldsymbol{PGI.c.rev}}\boldsymbol{)}$

$$P_{G6P}\left( {G6P}_{m+x}\left( t_{i}+\Delta t_{i} \right)-G6P_{m+x}\left( t_{i} \right) \right)=v_{PGI.p}\cdot{F6P}_{m+x}\left( t_{i} \right)\Delta t_{i}+v_{PGI.c}\cdot c^{I}\cdot{F6P}_{m+x}\left( t_{i} \right)\Delta t_{i}+v_{PGM.p.rev}\cdot{G1P}_{m+x}\left( t_{i} \right)\Delta t_{i}+v_{PGM.c.rev}\cdot c^{IV}\cdot{G1P}_{m+x}\left( t_{i} \right)\Delta t_{i}-\left( v_{PGM.p}+v_{PGI.p.rev} \right)\cdot{G6P}_{m+x}\left( t_{i} \right)\Delta t_{i}-\left( v_{PGM.c}+v_{PGI.c.rev} \right)\cdot c^{III}\cdot{G6P}_{m+x}\left( t_{i} \right)\Delta t_{i}$$

$\frac{\boldsymbol{dUDPG}}{\boldsymbol{dt}}\boldsymbol{=}\boldsymbol{v}_{\boldsymbol{GPU}}\boldsymbol{-}\boldsymbol{v}_{\boldsymbol{SPS}}$

$$P_{UDPG}\left( {UDPG}_{m+x}\left( t_{i}+\Delta t_{i} \right)-{UDPG}_{m+x}\left( t_{i} \right) \right)=v_{GPU}\cdot c^{IV}\cdot{G1P}_{m+x}\left( t_{i} \right)\Delta t_{i}-v_{SPS}\cdot{UDPG}_{m+x}\left( t_{i} \right)\Delta t_{i}$$

$\frac{\boldsymbol{dADPG}}{\boldsymbol{dt}}\boldsymbol{=}\boldsymbol{v}_{\boldsymbol{AGP}}\boldsymbol{-}\boldsymbol{v}_{\boldsymbol{SS}}$

$$P_{ADPG}\left( {ADPG}_{m+x}\left( t_{i}+\Delta t_{i} \right)-{ADPG}_{m+x}\left( t_{i} \right) \right)=v_{AGP}\cdot{G1P}_{m+x}\left( t_{i} \right)\Delta t_{i}-v_{SS}\cdot{ADPG}_{m+x}\left( t_{i} \right)\Delta t_{i}$$

# Supplementary Figures

**
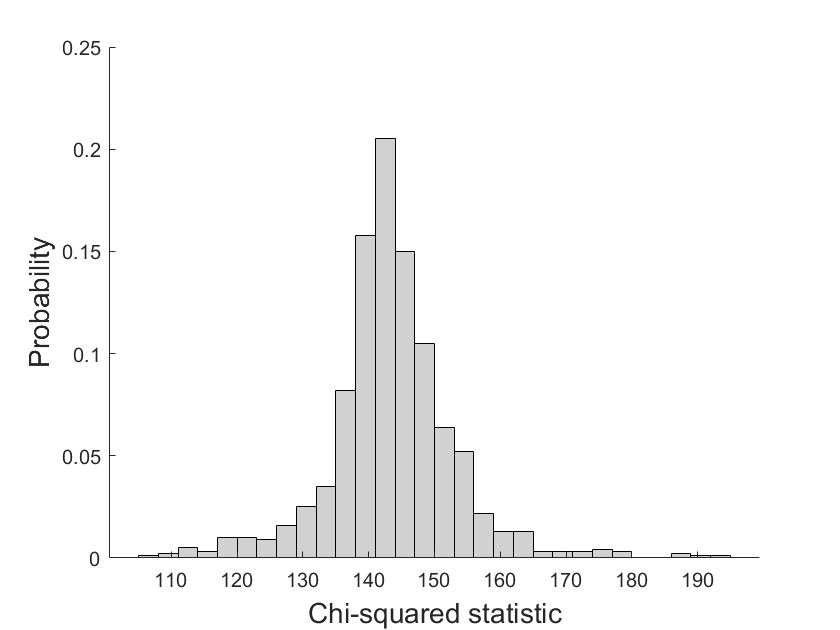
**

**Figure S1. Distribution of chi-squared statistic over 1000 bootstrap samples for *C. ohadii* under low light conditions.** The critical chi-square to accept model fit was 150 (118 degrees of freedom). chi-squared values for 81.6% of bootstrap samples were below the critical value.

**
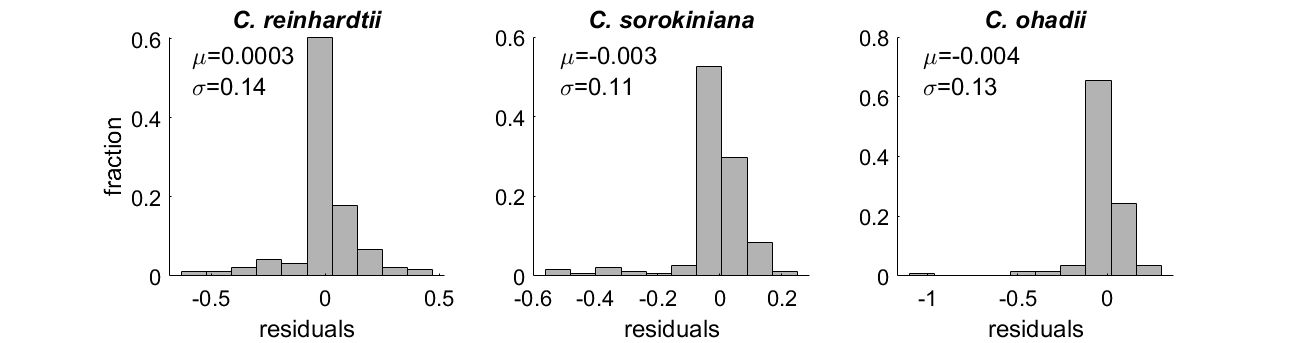
**

**Figure S2. Distribution of residuals obtained for fitting labeling dynamics using the ‘compartmented CBC model’ for three algae under LL with acceptable fit based on chi-squared statistic.**  All distributions were found to be normally distributed based on Kolmogorov-Smirnov test (p-value < 10^-20^).

**
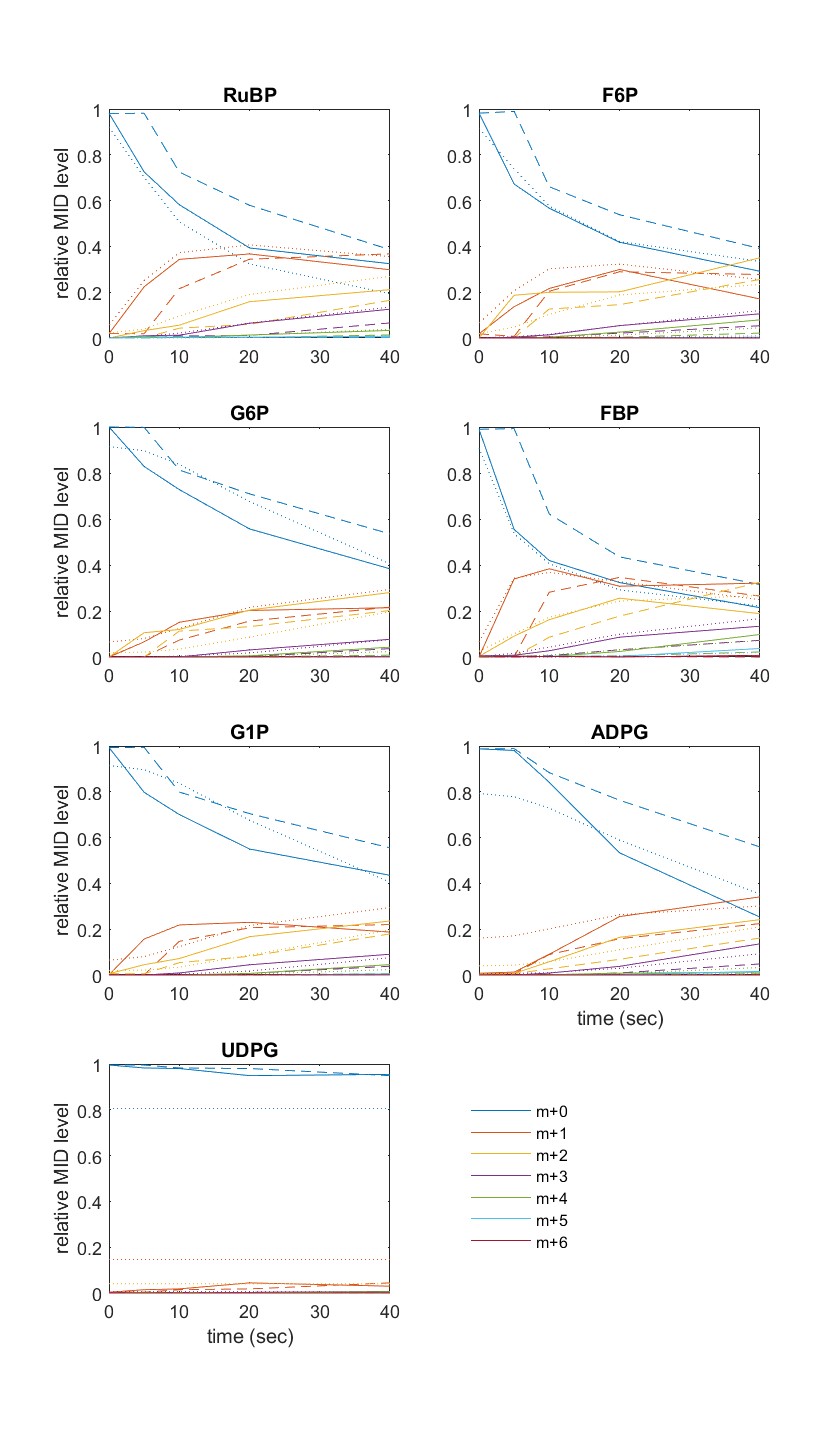
**

**Figure S3. Fitted labeling pattern obtained from INCA and SFCR for *C. reinhardtii*.** Relative labelling pattern measured (solid line) are compared to the fit obtained from INCA (dotted line) and SFCR (dashed line).

**
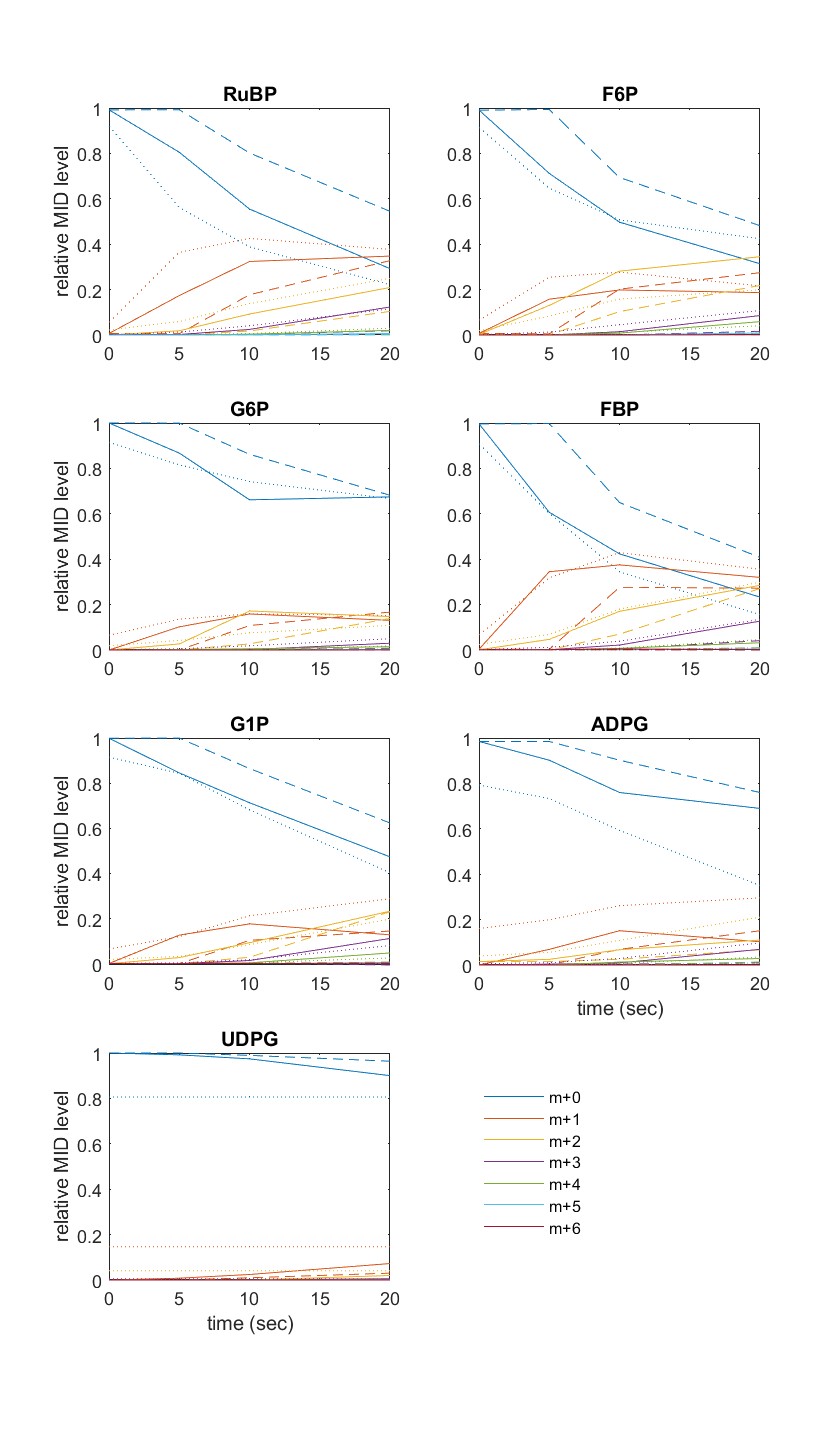
**

**Figure S4. Fitted labeling pattern obtained from INCA and SFCR for *C. sorokiniana*.** Relative labelling pattern measured (solid line) are compared to the fit obtained from INCA (dotted line) and SFCR (dashed line).

**
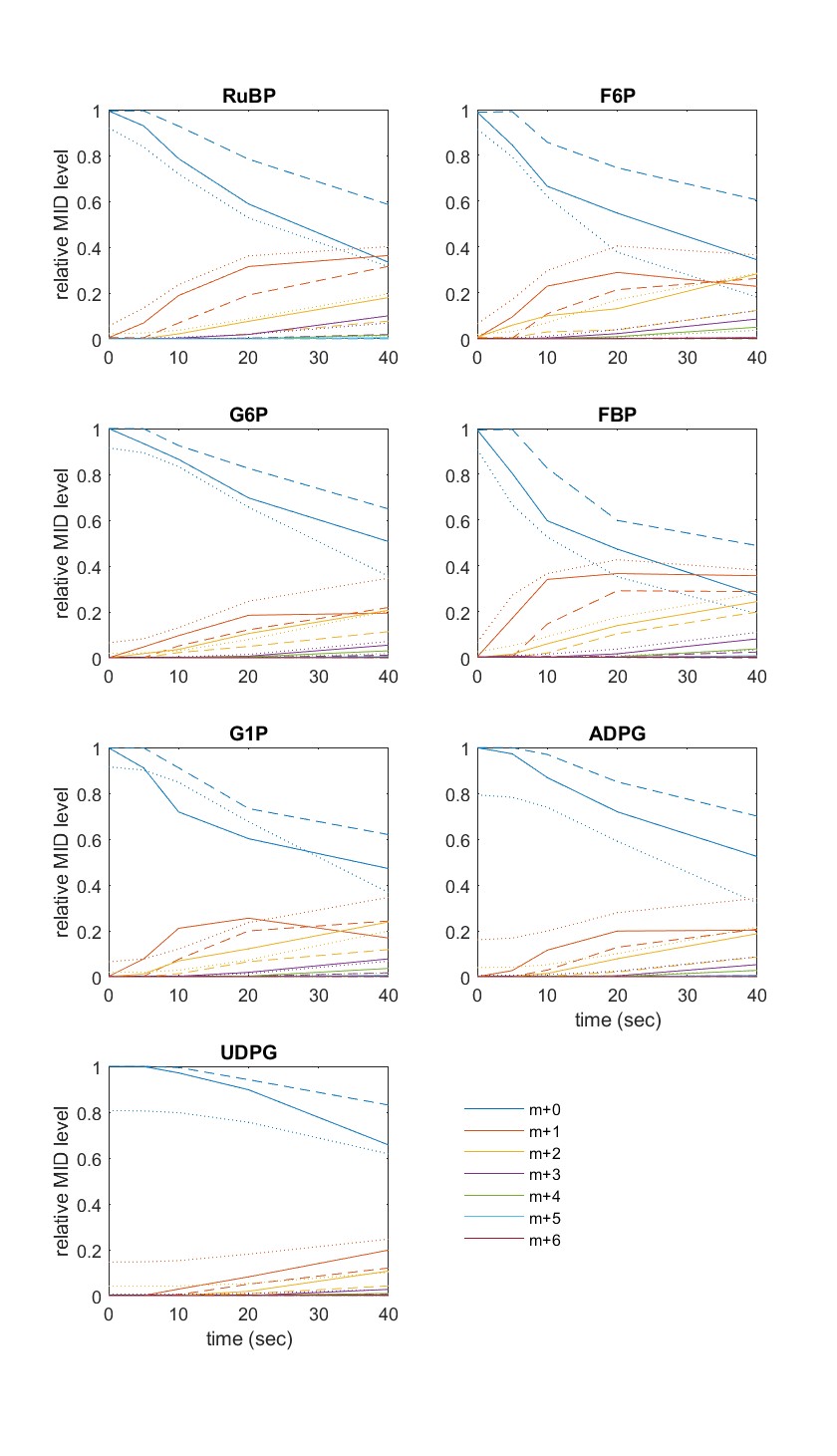
**

**Figure S5. Fitted labeling pattern obtained from INCA and SFCR for *C. ohadii* under LL condition.** Relative labelling pattern measured (solid line) are compared to the fit obtained from INCA (dotted line) and SFCR (dashed line).
